# Pathogenic mutations in the kinesin-3 motor KIF1A diminish force generation and movement through allosteric mechanisms

**DOI:** 10.1083/jcb.202004227

**Published:** 2021-01-26

**Authors:** Breane G. Budaitis, Shashank Jariwala, Lu Rao, Yang Yue, David Sept, Kristen J. Verhey, Arne Gennerich

**Affiliations:** 1 Program in Cellular and Molecular Biology, University of Michigan, Ann Arbor, MI; 2 Department of Computational Medicine and Bioinformatics, University of Michigan, Ann Arbor, MI; 3 Department of Biomedical Engineering, University of Michigan, Ann Arbor, MI; 4 Department of Cell and Developmental Biology, University of Michigan, Ann Arbor, MI; 5 Department of Anatomy and Structural Biology and Gruss-Lipper Biophotonics Center, Albert Einstein College of Medicine, New York, NY

## Abstract

The kinesin-3 motor KIF1A functions in neurons, where its fast and superprocessive motility facilitates long-distance transport, but little is known about its force-generating properties. Using optical tweezers, we demonstrate that KIF1A stalls at an opposing load of ~3 pN but more frequently detaches at lower forces. KIF1A rapidly reattaches to the microtubule to resume motion due to its class-specific K-loop, resulting in a unique clustering of force generation events. To test the importance of neck linker docking in KIF1A force generation, we introduced mutations linked to human neurodevelopmental disorders. Molecular dynamics simulations predict that V8M and Y89D mutations impair neck linker docking. Indeed, both mutations dramatically reduce the force generation of KIF1A but not the motor’s ability to rapidly reattach to the microtubule. Although both mutations relieve autoinhibition of the full-length motor, the mutant motors display decreased velocities, run lengths, and landing rates and delayed cargo transport in cells. These results advance our understanding of how mutations in KIF1A can manifest in disease.

## Teaser

Kinesin-3 motors are fast and superprocessive, but their force generation properties remain unclear. The authors show that KIF1A detaches under low opposing forces but rapidly reattaches to continue motility. Rapid reattachment depends on the class-specific K-loop, whereas mutations linked to neurodevelopmental disorders impair force generation and motility.

## Introduction

The cytoskeleton of eukaryotic cells forms the structural framework for fundamental cellular processes, including cell division, cell motility, intracellular trafficking, and cilia function. In most processes, the functional output of the microtubule (MT) cytoskeleton depends on a family of molecular motor proteins called kinesins. Kinesins are defined by the presence of a globular kinesin motor domain that contains sequences for binding ATP and MTs. Kinesins involved in intracellular trafficking use the energy of ATP hydrolysis for processive motility (ability to take multiple steps before dissociating) and force generation along the MT surface.

The kinesin-3 family is one of the largest among the kinesin superfamily, and its members are primarily involved in the anterograde transport of cargoes toward the plus ends of MTs in the periphery of the cell (Gabrych et al., 2019; Hirokawa et al., 2009; Siddiqui and Straube, 2017). Genetic and microscopy studies have implicated the kinesin-3 motor KIF1A and its orthologues in the transport of synaptic vesicle precursors and dense core vesicles to the axon terminal (Barkus et al., 2008; Hall and Hedgecock, 1991; Lo et al., 2011; Okada et al., 1995; Yonekawa et al., 1998; Zahn et al., 2004). A number of inherited variants and de novo mutations have been identified in human *KIF1A* from clinical studies. These mutations have been linked to neurodevelopmental and neurodegenerative disorders, including spastic paraplegias, encephalopathies, intellectual disability, autism, and sensory neuropathies (reviewed in Boyle et al., 2020; Gabrych et al., 2019; Guo et al., 2020; John et al., 2020; Nicita et al., 2020; Van Beusichem et al., 2020). In KIF1A-associated neurological disorder (KAND), the mutations span the entirety of the KIF1A protein sequence (Boyle et al., 2020). The majority are located within the core motor domain (aa 1–369) and are thus predicted to affect the motor’s motility properties, whereas mutations located outside the motor domain are likely involved in mediating dimerization, autoinhibition, and/or cargo binding (Gabrych et al., 2019).

Recent studies have shown that several members of the kinesin-3 family have striking motility properties, as they are exceptionally fast and superprocessive and have high MT landing rates (ability to productively engage with MTs; Hammond et al., 2009; Huckaba et al., 2011; Lessard et al., 2019; Okada et al., 1995; Rogers et al., 2001; Scarabelli et al., 2015; Siddiqui et al., 2019; Soppina et al., 2014; Tomishige et al., 2002; Zaniewski et al., 2020). However, little is known about the ability of kinesin-3 motors to generate and sustain force. A general understanding of how kinesin motors generate force is based largely on studies of kinesin-1 (Asbury et al., 2003; Brenner et al., 2020; Guydosh and Block, 2009; Hwang and Karplus, 2019; Ramaiya et al., 2017; Svoboda and Block, 1994), the founding member of the kinesin superfamily. Force generation requires the neck linker (NL), a flexible structural element that immediately follows the kinesin motor domain, which docks along the surface of the motor domain in response to ATP binding (Case et al., 2000; Hwang et al., 2017; Khalil et al., 2008; Rice et al., 1999). NL docking in kinesin-1 occurs in two steps. First is the “zippering” step, in which the first half of the NL (β9) interacts with β0 (the cover strand [CS]) of the core motor domain to generate a short β-sheet termed the cover-neck bundle (CNB; Hwang et al., 2008; Khalil et al., 2008). Although formation of the CNB has been observed in structures of motor domains from kinesin-3, kinesin-5, and kinesin-6 members (Atherton et al., 2014; Atherton et al., 2017; Goulet et al., 2012; Goulet et al., 2014; Hesse et al., 2013; Ren et al., 2018b; von Loeffelholz and Moores, 2019), its mechanical role in force generation has only been tested in kinesin-1 motors (Budaitis et al., 2019; Khalil et al., 2008). Second is the “latching” step, in which the second half of the NL (β10) interacts with surface residues of α1-β3 and β7 of the core motor domain and is latched in place via a conserved asparagine residue (the N-latch; Budaitis et al., 2019; Hwang et al., 2008; Khalil et al., 2008). A role for NL latching in force generation was recently demonstrated for kinesin-1 (Budaitis et al., 2019). Crystal structures of kinesin-3 motor domains suggest that close contact between α1-β3 and the NL may play a role in force generation for this family as well (Atherton et al., 2014; Nitta et al., 2008; Ren et al., 2018b).

Despite these structural similarities, several lines of evidence suggest that the force-generating properties of kinesin-3 motors may be different from those of other kinesin motors. First, when forced to compete with kinesin-1, KIF1A gives up easily, suggesting that it has a high load-dependent off-rate from the MT (Arpag et al., 2019; Arpag et al., 2014; Norris et al., 2014). Second, the *Caenorhabditis elegans* homologue UNC-104 displays a rapid decrease in velocity and increase in MT dissociation rate under load applied in an optical tweezers assay (Tomishige et al., 2002). Third, a short CNB was observed in crystal structures of the kinesin-3 KIF13B (Ren et al., 2018b). Here, we determined the force-generating properties of two members of the kinesin-3 family, the mammalian KIF1A motor and its homologue UNC-104, using a single-molecule optical tweezers assay (Nicholas et al., 2014). We show that these kinesin-3 motors stall at a maximal force of ~3 pN; however, they readily detach from the MT track rather than stall. Strikingly, KIF1A motors quickly reattach to the MT and resume force generation, leading to a sawtooth force generation pattern that is distinct from other kinesin motors to date. Rapid reattachment requires the class-specific and positively charged loop 12 (K-loop) of KIF1A.

To determine whether NL docking plays a critical role in force generation by KIF1A, we introduced disease-associated mutations based on their (a) location in structural elements predicted to be critical for NL docking and (b) mild disease phenotypes that suggest an impairment rather than loss of KIF1A protein activity. V8M and Y89D are de novo mutations that manifest in an autosomal dominant manner to cause pure hereditary spastic paraplegia with childhood onset (OMIM accession no. 610357; Iqbal et al., 2017) and neurodegeneration and spasticity with or without cerebellar atrophy or cortical visual impairment (OMIM accession no. 614255; ClinVar accession no. VCV000224157), respectively. The V8M mutation is located in β1, immediately following the CS, and may therefore prevent CNB formation. Notably, a valine in this position is highly conserved across the kinesin superfamily (Richard et al., 2016). The Y89D mutation is located at the α1-β3 intersection, and an aromatic residue (tyrosine or phenylalanine) at this position is highly conserved across the kinesin superfamily (Budaitis et al., 2019; Richard et al., 2016). Molecular dynamics (MD) simulations predict attenuating effects of both mutations on KIF1A force generation. In optical tweezers assays, both mutations resulted in a significant decrease in force output but had no effect on the motor’s ability to rapidly reengage with the MT track. In single-molecule fluorescence assays, both mutations resulted in decreases in speed, processivity, and landing rate on MTs under unloaded conditions. Finally, when working in teams in cells, the mutant motors show a significant delay in organelle transport. Collectively, our results support the proposed role for the NL as a mechanical element important for kinesin motors to transport against load. Our results also provide insight into how KAND-associated mutations affect KIF1A transport in cells.

## Results

### KIF1A and UNC-104 detach rather than stall under load

**Figure 1.**
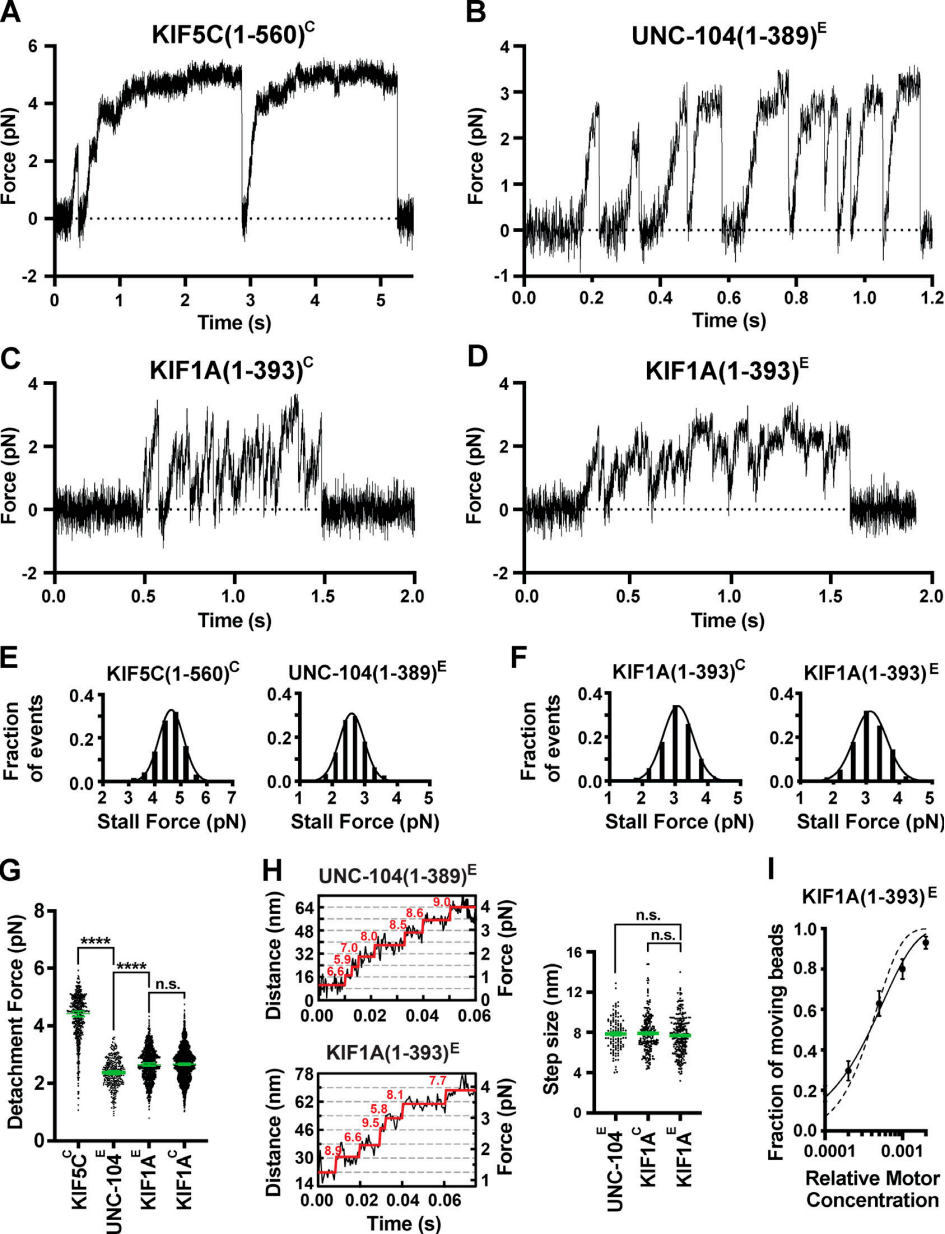
**Minimal dimeric KIF1A and UNC-104 motors detach under low force and rapidly reattach to the MT.** **(A–D)** Representative force versus time records of bead movement driven by single molecules of kinesin-1 KIF5C(1–560)^C^ (A), kinesin-3 UNC-104(1–389)^E^ (B), kinesin-3 KIF1A(1–393)^C^ (C), and kinesin-3 KIF1A(1–393)^E^ (D). **(E)** Stall force histograms of KIF5C(1–560)^C^ (4.64 ± 0.01 pN, mean ± SEM from Gaussian fit; stall plateaus ≥200 ms; *n* = 197) and UNC-104(1–389)^E^ (2.60 ± 0.01 pN, stall plateaus ≥10 ms; *n* = 163) compiling forces at *k* = 0.05–0.06 pN/nm from *n* = 3 and 2 repeated experiments, respectively. **(F)** As in E, but for KIF1A(1–393)^C^ (3.09 ± 0.01 pN, *n* = 418) and KIF1A(1–393)^E^ (3.12 ± 0.02 pN; *n* = 992) (*k* = 0.05–0.06 pN/nm; *n* = 4 and 4). **(G)** Detachment forces. Green bars indicate the median values with quartiles. KIF5C(1–560)^C^: 4.43 (3.79, 4.86) pN, *n* = 557; UNC-104(1–389)^E^: 2.37 (2.03, 2.70) pN, *n* = 355; KIF1A(1–393)^E^: 2.65 (2.25, 3.05) pN, *n* = 1,044; KIF1A(1–393)^C^: 2.66 (2.25, 3.01) pN, *n* = 1,912. Statistical significance was determined using an unpaired Welch’s *t* test (****, P < 0.0001). **(H)** Left, stepwise forward movements of UNC-104(1–389)^E^ (trap stiffness: *k* = 0.062 pN/nm; upper inset) and KIF1A(1–393)^E^ (*k* = 0.057 pN/nm; lower inset). The raw data are shown in black, and the steps detected by the step-finding program are shown in red. Right, measured step sizes. UNC-104(1–389)^E^: 7.9 ± 0.2 nm, mean ± SEM, *n* = 109; KIF1A(1–393)^E^: 7.7 ± 0.1 nm, *n* = 254; KIF1A(1–393)^C^: 7.9 ± 0.1 nm, *n* = 202. Statistical analysis using Welch’s *t* test shows that the step-size distributions are statistically indistinguishable. **(I)** Fraction of KIF1A(1–393)^E^-coated beads binding to and moving along MTs as a function of the relative motor concentration. The bead concentration was kept constant for all measurements, whereas the motor concentration was varied (*n* = 224 total number of beads tested; *n* = 70–100 at each concentration). The solid line represents the fit to the Poisson distribution 1 − exp(−λ*C*) for one or more motor molecules (processive model), where *C* is the relative motor concentration and λ is a fit parameter (*R*^2^ = 0.9886; Svoboda and Block, 1994). The dotted line represents the fit to the distribution 1 − exp(−λ*C*) − (λ*C*)exp(−λ*C*) for two or more molecules (nonprocessive model; *R*^2^ = 0.9023). Data values are displayed as the mean ± square root of [*f*(1 − *f*)/*n*], with *n* being the number of beads tested. n.s., not significant.

To examine the force output of kinesin-3 motors, we used optical tweezers with nanometer-level spatial resolution (Nicholas et al., 2014; Rao et al., 2019) to probe the force response of rat KIF1A and *C. elegans* UNC-104. We used the truncated versions KIF1A(1–393) and UNC-104(1–389), which dimerize via their native neck coil (α-helix 7) sequences, and appended a leucine zipper (LZ) sequence to maintain the dimer state as described previously (Soppina et al., 2014). KIF1A(1–393) in COS-7 cell lysates (referred to as KIF1A(1–393)^C^) was tagged with an AviTag and biotinylated by coexpressed BirA for attachment to streptavidin-coated trapping beads. KIF1A(1–393) purified from *Escherichia coli* cells (referred to as KIF1A(1–393)^E^) was tagged with SNAP_f_ (for labeling with tetramethylrhodamine [TMR]), EGFP (for attachment to anti-GFP–coated trapping beads), and 6×His tag for purification. UNC-104(1–389) purified from *E. coli* cells (referred to as UNC-104(1–389)^E^) was tagged with EGFP for attachment to anti-GFP–coated trapping beads and a 6×His tag for purification. As a control, we also performed experiments on the kinesin-1 motor KIF5C. Biotinylated KIF5C(1–560) motors in COS-7 cell lysates (referred to as KIF5C(1–560)^C^) displayed typical force-generating events in which stalling occurred at an average force of 4.6 ± 0.04 pN (motor stalls ≥200 ms; mean ± SEM; Fig. 1, A and E), but the motor often detached from the MT at a smaller average force (4.3 ± 0.04 pN, mean ± SEM; Fig. 1 G and Table 1), consistent with previous studies (Brenner et al., 2020; Budaitis et al., 2019; Khalil et al., 2008; Svoboda and Block, 1994).

**Table 1.** Single-molecule detachment forces

Kinesin motor		COS-7 cell lysate (pN)	*E. coli* expressed (pN)
Kinesin-1	KIF5C(1–560)^C^	4.4 (3.8, 4.9)	ND
Kinesin-3	UNC-104(1–389)^E^	ND	2.4 (2.0, 2.7)
	KIF1A(1–393)	2.7 (2.3, 3.0)	2.7 (2.3, 3.1)
	KIF1A(1–393)-SW^E^	ND	2.0 (1.6, 2.3)
	KIF1A(1–393)-SWA^E^	ND	1.8 (1.5, 2.1)
	KIF1A(1–393)-V8M	2.0 (1.7, 2.2)	1.9 (1.7, 2.2)
	KIF1A(1–393)-Y89D	1.0 (0.9, 1.2)	1.0 (0.9, 1.2)
	KIF1A(FL^Act^)^C^	2.8 (2.4, 3.3)	ND
	KIF1A(FL^Act^)-V8M^C^	1.8 (1.5, 2.1)	ND
	KIF1A(FL^Act^)-Y89D^C^	1.1 (0.9, 1.3)	ND

Data are reported as median (quartiles). ^C^, COS-7 expression; ^E^, *E. coli* expression.

**Figure S1.**
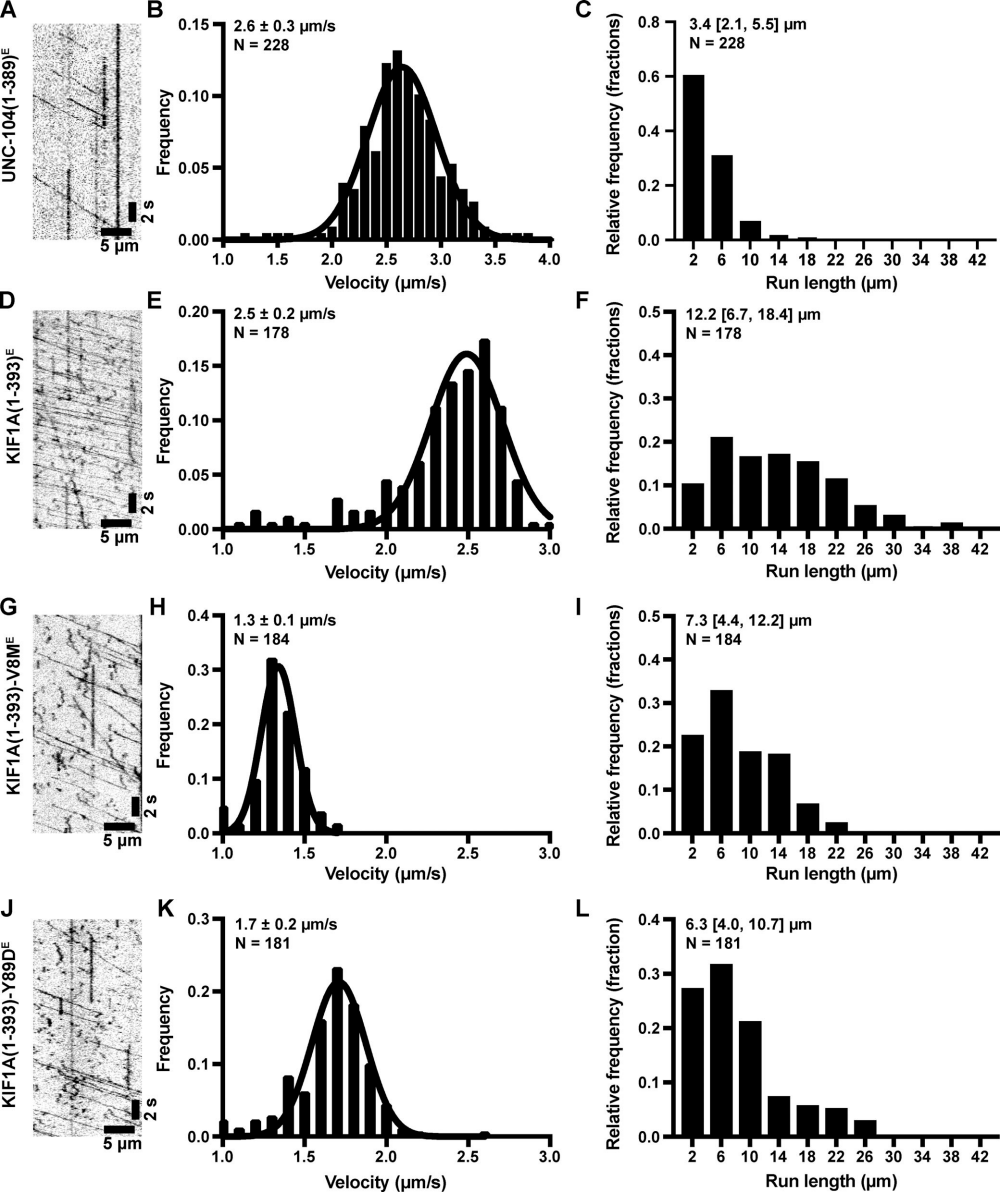
**Velocity and processivity of *E. coli*–expressed and TMR-labeled UNC-104 and WT and mutant KIF1A.** **(A)** Example kymograph of UNC-104(1–393)^E^ purified from *E. coli* bacteria. **(B and C) **From the kymographs, single-motor velocities (B) and run lengths (C) were determined. The mean values ± SEM (for velocities) and the median with quartiles (for run lengths) are indicated on each graph. **(D–F)** As in A–C, but for WT KIF1A(1–393)^E^. **(G–I)** As in A–C, but for the KIF1A(1–393)-V8M^E^ mutant. **(J–L)** As in A–C, but for the KIF1A(1–393)-Y89D^E^ mutant.

**Figure S2.**
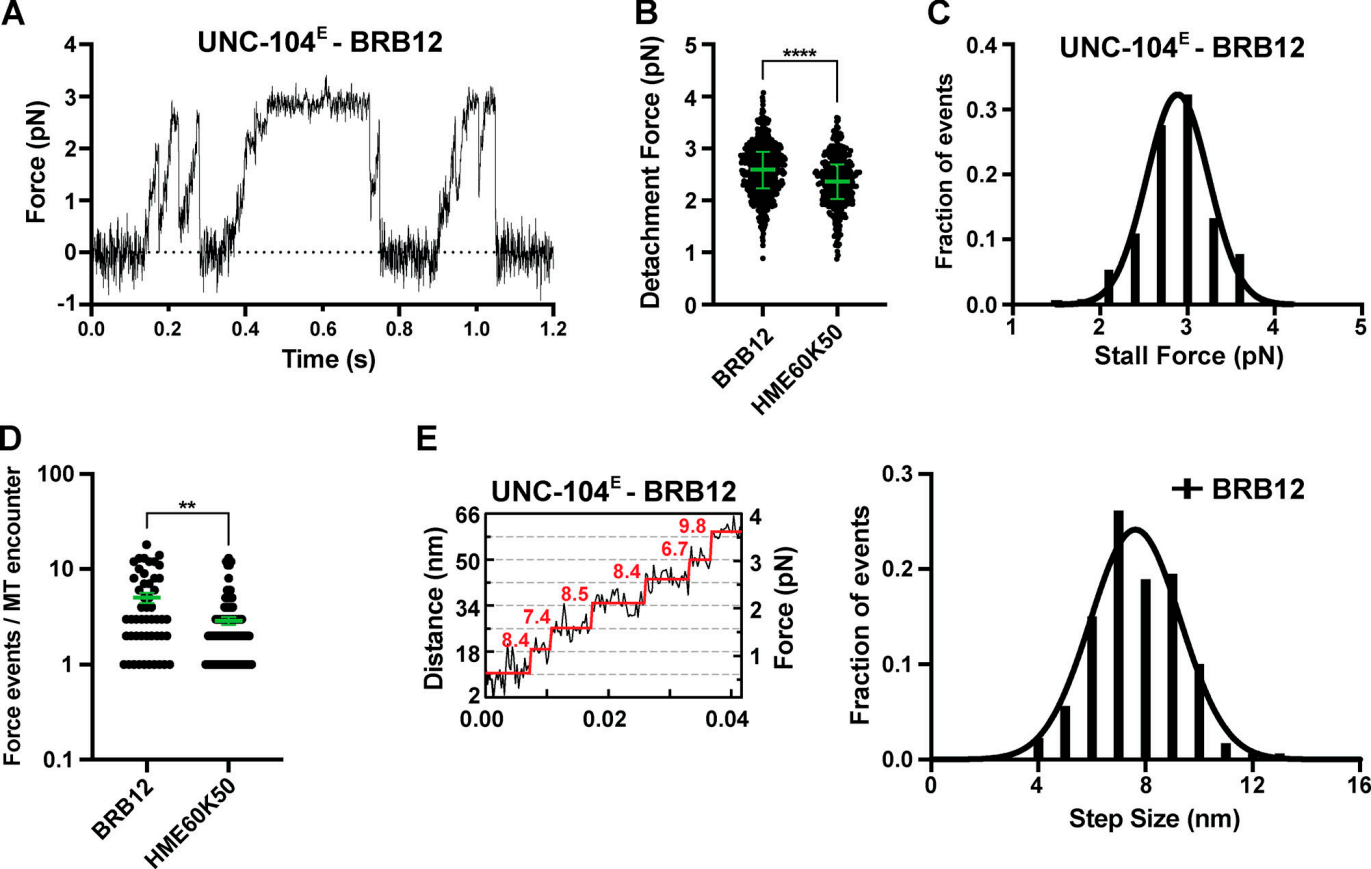
**Analysis of UNC-104 force generation in BRB12 buffer.** **(A)** Representative force versus time record of bead movement driven by a single molecule of UNC-104(1–389)^E^. **(B)** UNC-104(1–389)^E^ detachment forces measured in BRB12 and HME60K50 (reproduced from Fig. 1 G for comparison). Green bars in B and D indicate the median values with quartiles. UNC-104(1–389)^E^ in BRB12: 2.59 (2.23, 2.94) pN, *n* = 561; UNC-104(1–389)^E^ in HME60K50: 2.37 (2.03, 2.70) pN, *n* = 355. Statistical significance was determined by using an unpaired Welch’s *t* test (****, P < 0.0001). **(C)** Stall force histogram of UNC-104(1–389)^E^ in BRB12 (2.89 ± 0.02 pN, mean ± SEM from Gaussian fit; stall plateaus ≥10 ms; *n* = 126) compiling forces at *k* = 0.05–0.06 pN/nm. **(D)** Number of engagement events per MT encounter. UNC-104(1–389)^E^ in BRB12: 5.0 ± 0.6 (mean ± SEM, *n* = 50); UNC-104(1–389)^E^ in HME60K50: 2.9 ± 0.3 (mean ± SEM, *n* = 92). **, P < 0.0022. **(E)** Left, stepwise forward movements of UNC-104(1–389)^E^ in BRB12 (trap stiffness: *k* = 0.06 pN/nm). The raw data are shown in black, and the steps detected by the step-finding program are shown in red. Right, measured step sizes: 7.6 ± 0.1 nm (mean ± SEM from Gaussian fit; *n* = 180).

Individual UNC-104(1–389)^E^ motors were processive in the absence of load (Fig. S1, A–C) and frequently detached under load before reaching a stall plateau (Fig. 1 B). In HME60K50 buffer at pH 7.2, UNC-104(1–389)^E^ motors stalled (≥10-ms criterion) at 2.6 ± 0.01 pN (mean ± SEM; Fig. 1 E) but detached at a force of 2.4 (2.0, 2.7) pN (median [quartiles]; Fig. 1 G and Table 1). In the more frequently used BRB12 buffer at pH 6.8 (Tomishige et al., 2002), the stall force and the average detachment force of UNC-104^E^ increased to 2.9 ± 0.02 pN (mean ± SEM) and 2.6 (2.2, 2.9) pN (median [quartiles]), respectively (Fig. S2, A–C).

**Figure S3.**
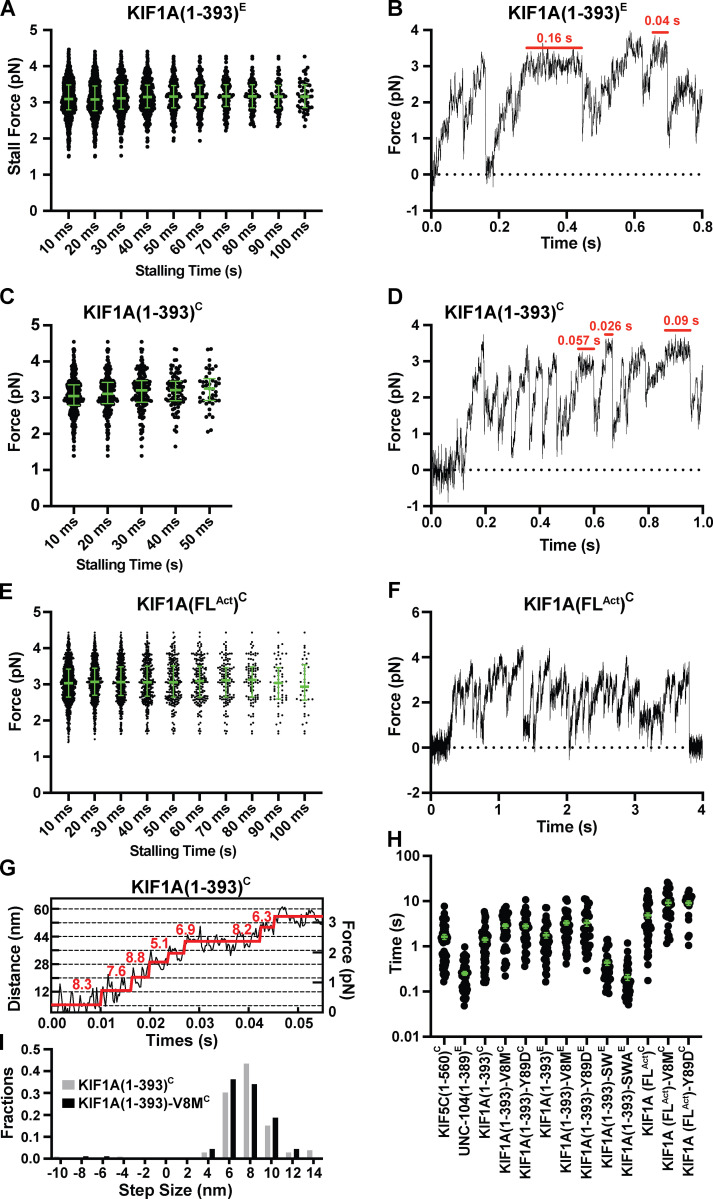
**Additional stall force, step size, and cluster length analyses.** **(A)** Maximal force (“stall force”) sustained by KIF1A(1–393)^E^ in HME60K50 during a single run against load for a minimum time duration of 10 ms (3.1 [2.8, 3.5] pN, *n* = 992), 20 ms (3.1 [2.8, 3.5] pN, *n* = 699), 30 ms (3.1 [2.8, 3.5] pN, *n* = 452), 40 ms (3.1 [2.8, 3.5] pN, *n* = 310), 50 ms (3.2 [2.9, 3.5] pN, *n* = 220), 60 ms (3.2 [2.9, 3.5] pN, *n* = 159), 70 ms (3.2 [2.9, 3.5] pN, *n* = 118), 80 ms (3.2 [2.9, 3.5] pN, *n* = 88), 90 ms (3.2 [2.9, 3.5] pN, *n* = 67), and 100 ms (3.2 [2.9, 3.5] pN, *n* = 50), respectively. **(B)** Force versus time record of bead movement driven by a single molecule of KIF1A(1–389)^E^ in HME60K50 (*k =* 0.056 pN/nm). Stalling events (red horizontal bars) can be observed but are rare. **(C and D)** As in A and B, but for WT KIF1A(1–393)^C^ (*k =* 0.058 pN/nm): 10 ms (3.0 [2.8, 3.4] pN, *n* = 418), 20 ms (3.1 [2.8, 3.4] pN, *n* = 221), 30 ms (3.2 [2.9, 3.5] pN, *n* = 138), 40 ms (3.2 [2.9, 3.5] pN, *n* = 82), and 50 ms (3.3 [2.9, 3.5] pN, *n* = 43). **(E)** As in A, but for KIF1A(FL^Act^)^C^: 10 ms (3.0 [2.6, 3.4] pN, *n* = 1,019), 20 ms (3.1 [2.7, 3.5] pN, *n* = 718), 30 ms (3.1 [2.7, 3.5] pN, *n* = 480), 40 ms (3.1 [2.7, 3.5] pN, *n* = 330), 50 ms (3.1 [2.6, 3.5] pN, *n* = 216), 60 ms [3.1 (2.6, 3.5) pN, *n* = 163], 70 ms [3.1 (2.6, 3.5) pN, *n* = 117], 80 ms [3.1 (2.6, 3.5) pN, *n* = 86], 90 ms [3.0 (2.6, 3.5) pN, *n* = 57], and 100 ms [2.9 (2.6, 3.5) pN, *n* = 39]. **(F)** Force versus time record of bead movement driven by a single molecule of KIF1A(FL^Act^)^C^ in HME60K50 (*k =* 0.056 pN/nm). **(G)** Stepwise forward movements of KIF1A(1–393)^C^ in HME60K50 (trap stiffness: *k* = 0.057 pN/nm). The raw data are shown in black, and the steps detected by the step-finding program are shown in red. **(H)** Duration of clusters of force generation events per MT encounter for all constructs measured in HME60K50. Green bars in A, C, E, and H indicate the median values with quartiles. KIF5C(1–560)^C^ (1.3 [0.5, 2.1] pN, *n* = 55), UNC-104(1–389)^E^ (0.21 [0.11, 0.32] pN, *n* = 52), KIF1A(1–393)^C^ (1.1 [0.5, 1.7] pN, *n* = 61), KIF1A(1–393)-V8M^C^ (2.5 [0.9, 4.7] pN, *n* = 42), KIF1A(1–393)-Y89D^C^ (2.5 [1.1, 4.0] pN, *n* = 43), KIF1A(1–393)^E^ (1.3 [0.7, 2.4] pN, *n* = 43), KIF1A(1–393)-V8M^E^ (2.8 [1.6, 4.2] pN, *n* = 46), KIF1A(1–393)-Y89D^E^ (2.3 [1.0, 5.4] pN, *n* = 34), KIF1A(1–393)-SW^E^ (0.31 [0.19, 0.48] pN, *n* = 39), KIF1A(1–393)-SWA^E^ (0.16 [0.08, 0.25] pN, *n* = 47), KIF1A(FL^Act^)^C^ (2.9 [1.6, 7.7] pN, *n* = 46), KIF1A(FL^Act^)-V8M^C^ (7.4 [4.2, 13.9] pN, *n* = 28), and KIF1A(FL^Act^)-Y89D^C^ (9.7 [4.8, 12.2] pN, *n* = 19). **(I)** Analysis of the steps taken against loads of 1–2 pN by WT KIF1A(1–393)^C^ and KIF1A(1–393)-V8M^C^ compiling data at *k* = 0.05–0.06 pN/nm. Measure forward step sizes: 7.9 ± 0.2 nm (KIF1A(1–393)^C^; *n* = 106) and 7.7 ± 0.2 nm (KIF1A(1–393)-V8M^C^; *n* = 91).

Individual KIF1A motors also underwent fast motility in the absence of load (Fig. S1, D–F; and Fig. 8) and rapidly detached from the MT when subjected to force (Fig. 1, C and D). KIF1A(1–393)^C^ and KIF1A(1–393)^E^ stalled at similar forces (3.09 ± 0.01 pN and 3.12 ± 0.02 pN [mean ± SEM], respectively; ≥10-ms criterion; Fig. 1 F and Fig. S3, A–D) but frequently detached before stalling (2.7 [2.3, 3.0] pN and 2.7 [2.3, 3.1] pN, median [quartiles]; Fig. 1 G and Table 1). The similarities in stall forces and detachment forces between KIF1A and UNC-104 demonstrates that these kinesin-3 motors have similar force generation capabilities. In addition, both KIF1A and UNC-104 motors take load-independent steps of ~8 nm (Fig. 1 H, S2 E, and S3 G). Thus, although the ability of these kinesin-3 motors to generate force is diminished as compared with that of kinesin-1 motors, KIF1A and UNC-104 also take similar center-of-mass steps along MTs under load.

**Figure 2.**
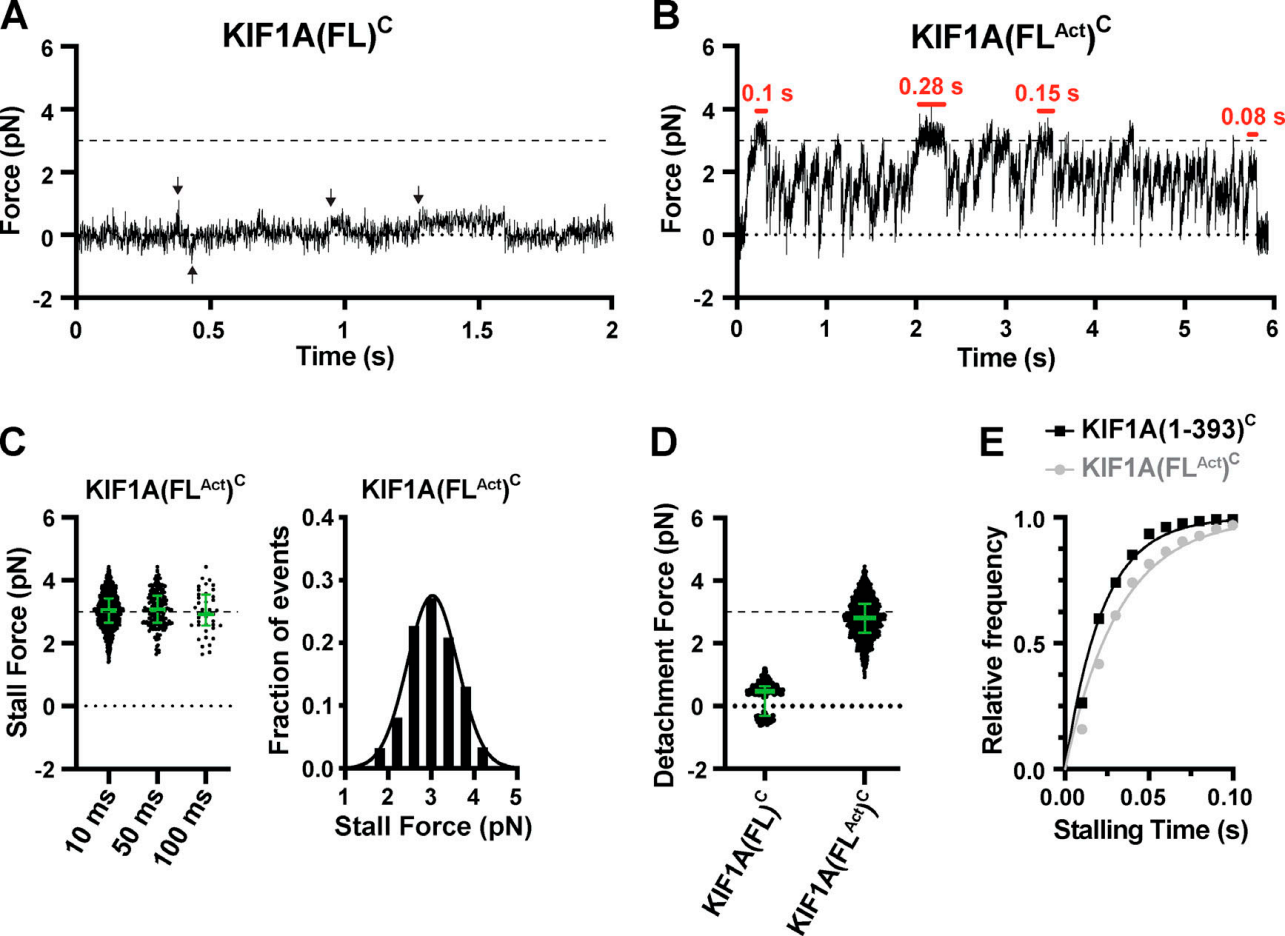
**FL KIF1A stalls at a force of 3 pN.** **(A)** Representative force versus time record of bead movement driven by KIF1A(FL)^C^ at 1 mM ATP and *k* = 0.049 pN/nm. Black arrows, events counted as force generation events. **(B)** Representative force versus time record of bead movement driven by KIF1A(FL^Act^)^C^. Red horizontal bars, stalling events. *k* = 0.056 pN/nm. **(C)** Left, stall force distributions for KIF1A(FL^Act^)^C^ as a function of the stalling criterion (minimum stalling time). 10 ms: 3.03 ± 0.02 pN, *n* = 1,019; 50 ms: 3.08 ± 0.04 pN, *n* = 216; 100 ms: 2.98 ± 0.11 pN, *n* = 39. Right, stall force histogram for the 10-ms stalling criterion (3.03 ± 0.03 pN, mean ± SEM from Gaussian fit) across two independent experiments. **(D)** Detachment forces. The green bars in C and D indicate the median value with quartiles. For KIF1A(FL)^C^, analysis of forces generated above the detection limit of ~0.3 pN reveals only weak force generation events up to ~1 pN in the MT plus end direction and <0.5 pN forces in both directions due to diffusional MT encounters. KIF1A(FL^Act^)^C^: 2.82 (2.35, 3.27) pN, *n* = 1,433 across two independent experiments. **(E)** Cumulative distributions of the measured stalling times of KIF1A(1–393)^C^ (black squares) and KIF1A(FL^Act^)^C^ (gray circles). The solid lines represent the fits to the cumulative distribution function (CDF) 1 − exp(−t/*T*), with *T* giving average (characteristic) stalling times of KIF1A(1–393)^C^: 22.3 ± 0.7 ms (mean ± SEM) and KIF1A(FL^Act^)^C^: 32.2 ± 0.9 ms.

To ensure that our minimal dimeric version of KIF1A(1–393) reflects the behavior of the full-length (FL) motor, we performed similar optical trapping experiments with biotinylated FL rat KIF1A in COS-7 cell lysates (KIF1A(FL)^C^). While FL KIF1A assumes an autoinhibited state in the absence of cargo (Hammond et al., 2009; Okada et al., 1995; Soppina et al., 2014), we hypothesized that the attachment of KIF1A’s biotinylated C-terminal tail to trapping beads would relieve autoinhibition, as has been observed for other kinesin motors (Imanishi et al., 2006; Svoboda and Block, 1994). However, KIF1A(FL) exhibited only weak (<1 pN) force generation events toward the MT plus end as well as diffusional encounters that resulted in small displacements (up to 0.5 pN) in both MT directions (Fig. 2 A). We therefore concluded that bead attachment alone is not sufficient to activate FL KIF1A. We thus took advantage of previous work demonstrating that a single point mutation (V483N) in the first coiled-coil domain of KIF1A relieves the motor’s auto-inhibitory conformation (Huo et al., 2012; Soppina et al., 2014; Yue et al., 2013). Here, we refer to this constitutively active FL motor as KIF1A(FL^Act^)^C^. Single KIF1A(FL^Act^)^C^ motors stalled (≥10-ms criterion) at 3.03 ± 0.03 pN (mean ± SEM; Fig. 2, B and C; and Fig. S3 E), similar to the minimal dimeric motor KIF1A(1–393)^C^ (Fig. 1 F; P < 0.41, Welch’s *t* test).

Interestingly, the KIF1A(FL^Act^)^C^ motors detached at a higher average force (2.8 [2.4, 3.3] pN, median [quartiles], Fig. 2 D) than the minimal dimeric motor (Fig. 1 G and Table 1; P < 0.0001, unpaired Welch’s *t* test) and stalled for a longer time (32.0 ± 0.9 ms, mean ± SEM; Fig. 2 E) than the minimal dimeric motor (22.0 ± 0.7 ms; Fig. 2 E; P < 0.0001, Welch’s *t* test). Recent work demonstrated that for kinesin-1, smaller vertical forces result in larger detachment forces and an increased likelihood of stalling (Khataee and Howard, 2019; Pyrpassopoulos et al., 2020). We surmise that a similar effect occurs for the longer KIF1A(FL^Act^)^C^ motor, which forms a smaller angle with the MT surface than the minimal dimeric motor and thus undergoes more frequent and longer stalls. In conclusion, both minimal dimeric and FL KIF1A motors display similar stall forces (~3 pN) as well as detachment forces (~2.7 pN; Table 1).

### The K-loop enables rapid rebinding of KIF1A to MTs and a clustering of force generation events

**Figure 3.**
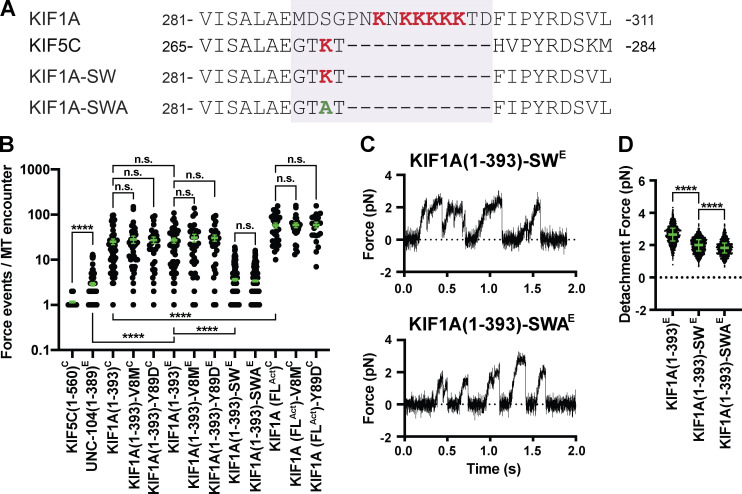
**The K-loop of KIF1A contributes to the clustering of force-generation events.** **(A)** Sequence alignment of KIF1A and KIF5C and sequences of the KIF1A(1–393)-SW and KIF1A(1–393)-SWA mutants. Purple box, loop 12. **(B)** Number of engagement events per MT encounter (averages during the clustering events per MT encounter are shown in Fig. S3 H). KIF5C(1–560)^C^: 1.2 ± 0.1 (mean ± SEM; *n* = 50); UNC-104(1–389)^E^: 2.9 ± 0.3 (*n* = 92); KIF1A(1–393)^C^: 25 ± 4 (*n* = 50); KIF1A(1–393)-V8M^C^: 28 ± 5 (*n* = 38); KIF1A(1–393)-Y89D^C^: 27 ± 4 (*n* = 31); KIF1A(1–393)^E^: 27 ± 3 (*n* = 50); KIF1A(1–393)-V8M^E^: 31 ± 6 (*n* = 30); KIF1A(1–393)-Y89D^E^: 31 ± 5 (*n* = 30); KIF1A(1–393)-SW^E^: 3.6 ± 0.2 (*n* = 234); KIF1A(1–393)-SWA^E^: 3.4 ± 0.2 (*n* = 286); KIF1A(FL^Act^)^C^: 58 ± 7 (*n* = 30); KIF1A(FL^Act^)-V8M^C^: 59 ± 7 (*n* = 24); KIF1A(FL^Act^)-Y89D^C^: 59 ± 8 (*n* = 20) across two to five independent experiments. ****, P < 0.0001 by unpaired Welch’s *t* test. **(C)** Representative force versus time records of bead movement driven by single molecules of KIF1A(1–393)-SW^E^ (top, *k* = 0.041 pN/nm) and KIF1A(1–393)-SWA^E^ (bottom, *k* = 0.042 pN/nm). **(D)** Detachment forces. Green bars in B and D indicate the median values with quartiles. KIF1A(1–393)^E^: 2.65 (2.25, 3.05) pN, *n* = 1,044 (from Fig. 1 G and included here for comparison); KIF1A(1–393)-SW^E^: 2.00 (1.64, 2.34) pN, *n* = 1,158; KIF1A(1–393)-SWA^E^: 1.84 (1.51, 2.14) pN, *n* = 1,214 across two to five independent experiments. ****, P < 0.0001 by Welch’s *t* test. n.s., not significant.

One striking observation was the ability of UNC-104 and KIF1A motors to frequently reengage with the MT track after load-induced detachment. Whereas kinesin-1 KIF5C(1–560)^C^ exhibited only 1.2 ± 0.1 force generation events per MT encounter, UNC-104(1–389)^E^ produced 2.9 ± 0.3 events, KIF1A(1–393)^E^ produced 27 ± 3 events, KIF1A(1–393)^C^ produced 25 ± 4 events, and KIF1A(FL^Act^)^C^ produced 58 ± 7 events (mean ± SEM; Figs. 3 B and S2 D). Here, an MT encounter is defined as the time period during which the bead is actively pulled away from the trap center, while the number of force generation events per MT encounter defines the number of times that the motor pulls the bead forward, releases, rebinds, and moves forward again before the bead is pulled back to the trap center. These rapid detachment and reattachment cycles result in a “clustering” of force generation events (Fig. 1, B–D; Fig. 2 B; and Fig. S3, B, D, and F).

**Figure S4.**
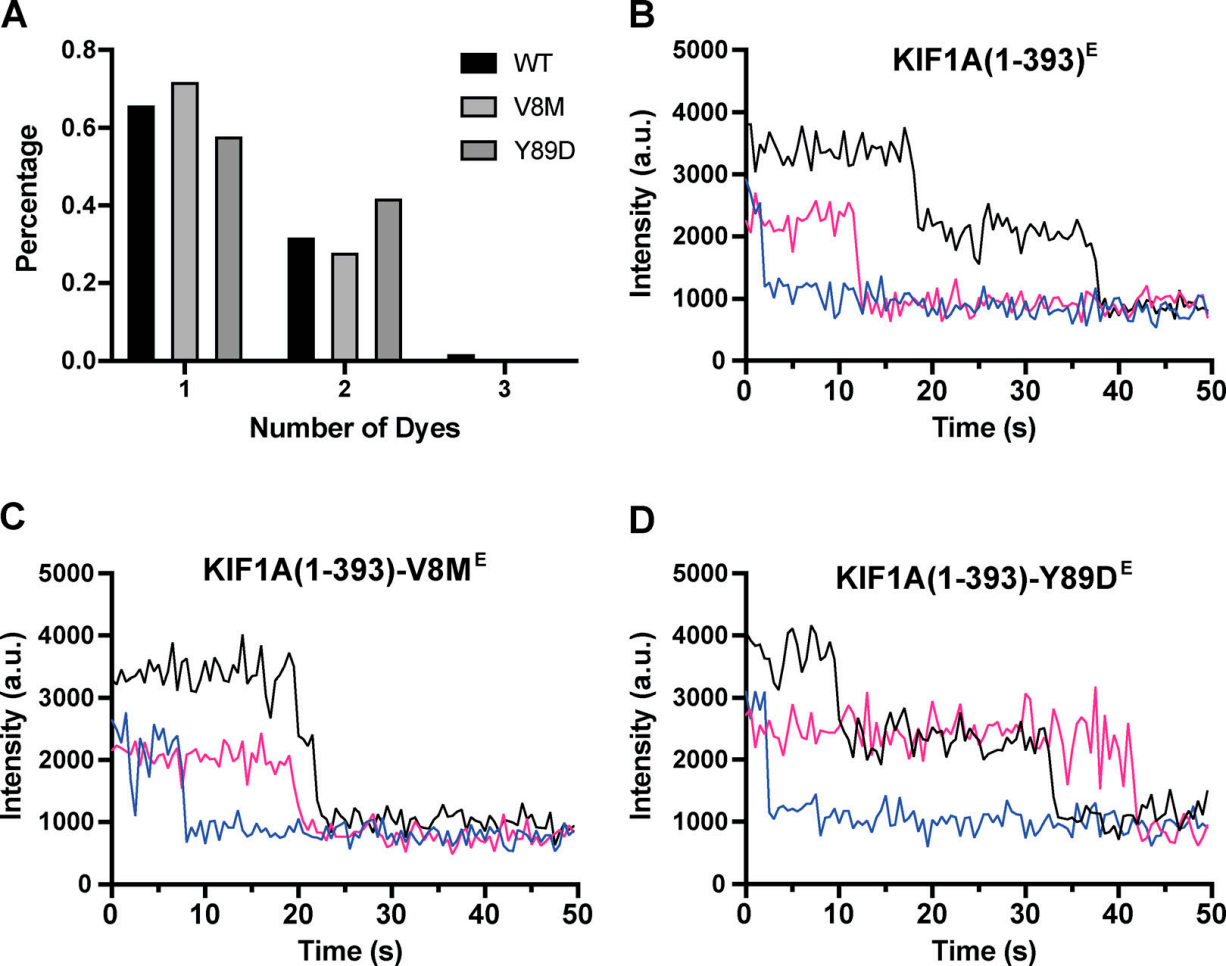
**Photobleaching analysis of TMR-labeled KIF1A(1–393)^E^ and mutants.** **(A)** Percentage of one-step, two-step, and three-step photobleaching events for KIF1A(1–393)^E^, KIF1A(1–393)-V8M^E^, and KIF1A(1–393)-Y89D^E^. **(B)** Representative examples of photobleaching events for WT KIF1A(1–393)^E^. **(C)** As in B, but for KIF1A(1–393)-V8M^E^. **(D)** As in B, but for KIF1A(1–393)-Y89D^E^.

As such a sawtooth force generation pattern has not been reported for a kinesin motor, we first wanted to rule out that the clustering of force generation events was caused by the activities of multiple KIF1A motors bound to the same bead. To do so, we analyzed the fraction of force-generating beads as a function of KIF1A(1–393)^E^ motor concentration and determined that the data are best described by a model that accounts for force generation by one or more motors (“processive” model), as opposed to a model for two or more motors (“non-processive” model; Fig. 1 I). To ensure that our measurements were performed at the single-molecule level, we used motor dilutions at which the fraction of beads moving was ≤0.3 as described previously (Brenner et al., 2020). In addition, photobleaching analysis of fluorescently tagged KIF1A(1–393)^C^ and KIF1A(1–393)^E^ motors further excludes the possibility of motor aggregation (Fig. S4; Hammond et al., 2009; Soppina et al., 2014). Thus, single KIF1A motors have the ability to rapidly reattach to the MT and resume motion after detachment, a property that gives rise to a sawtooth force generation pattern.

We therefore tested the hypothesis that KIF1A’s class-specific positively charged loop 12 (the K-loop) contributes to rapid rebinding to the MT track. This hypothesis is based on our previous work showing that the K-loop is responsible for KIF1A’s high on-rate toward MTs under unloaded conditions (Lessard et al., 2019; Soppina and Verhey, 2014). To test this hypothesis, we created a “swap” mutant in which KIF1A’s loop 12 was replaced with that of kinesin-1 (KIF1A(1–393)-SW^E^; Fig. 3 A). We also created a swap construct in which the remaining lysine was mutated to alanine (KIF1A(1–393)-SWA^E^; Fig. 3 A). Both the KIF1A(1–393)-SW^E^ and KIF1A(1–393)-SWA^E^ motors showed a significant reduction (P < 0.0001, Welch’s *t* test) in the average number of rebinding events per MT encounter (3.6 ± 0.2 and 3.4 ± 0.2 [mean ± SEM], respectively; Fig. 3, B and C) compared with WT KIF1A(1–393). These results demonstrate that the class-specific K-loop plays an important role in rapid rebinding of KIF1A to the MT. In addition, we found that both KIF1A(1–393)-SW^E^ and KIF1A(1–393)-SWA^E^ motors display significantly reduced (P < 0.0001, Welch’s *t* test) average detachment forces (2 [1.6, 2.3] pN (median [quartiles]) and 1.8 [1.5, 2.1] pN, respectively; Fig. 3 D) compared with the WT motor (Fig. 3 D and Table 1), suggesting that the K-loop also contributes to the motor’s ability to stay bound to the MT under load.

### KIF1A disease variants are predicted to impact motor force generation

**Figure 4.**
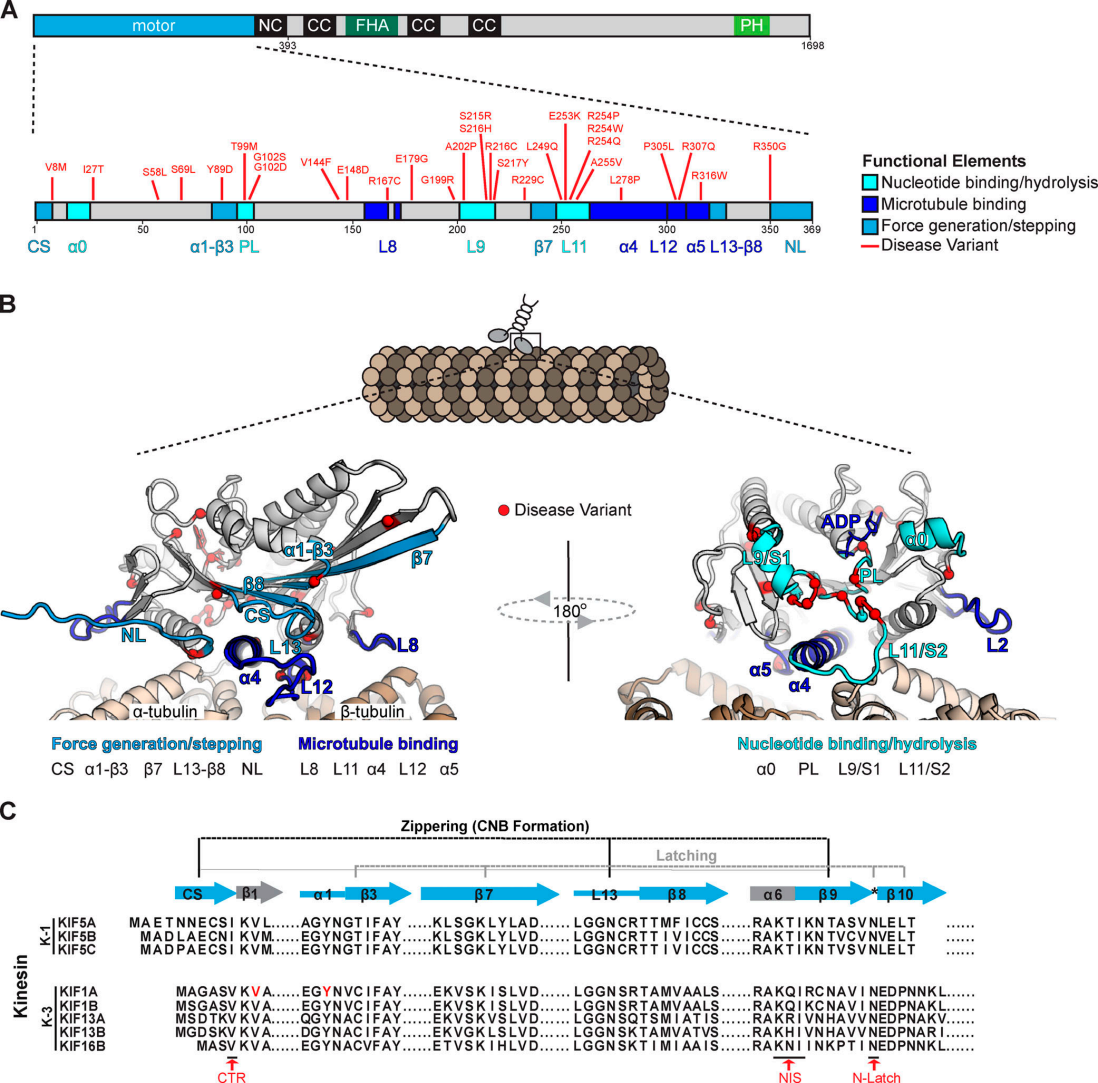
**KIF1A disease variants cluster within regions of the motor domain critical for MT binding, nucleotide binding/hydrolysis, and stepping/force generation.** **(A)** Schematic of domain structure of FL KIF1A and location of disease variants (red) within the KIF1A motor domain. **(B)** Ribbon representation of the KIF1A motor domain in the ADP-bound, tubulin-bound state (PDB accession no. 4UY0). Functional elements: dark blue, MT binding (loop 8, α4, loop 12, α5); medium blue, stepping/force generation (CS, α1-β3, β8, loop 13, NL); and cyan, nucleotide binding/hydrolysis (loop 9, loop 11, P loop, α0). **(C)** Alignment of sequences implicated in force generation for kinesin-1 and kinesin-3 families. NC, neck coil; CC, coiled-coil; FHA, forkhead associated; PH, pleckstrin homology; CTR, coverstrand terminal residue; NIS, NL initiation sequence.

We hypothesized that the mechanism of KIF1A force generation is similar to that of kinesin-1 and uses nucleotide-dependent conformational changes of the NL. To test this, we looked for KAND-associated mutations located in regions predicted to be critical for NL docking. We mapped KAND-associated mutations onto the protein sequence (Fig. 4 A, red lines) and structure (Fig. 4 B, red circles) of the KIF1A motor domain (Protein Data Bank [PDB] accession no. 4UY0; Atherton et al., 2014). The majority of KAND-associated mutations cluster within functional elements critical for MT binding, nucleotide binding, or force generation (Fig. 4, A and B; and Table 2; Boyle et al., 2020). We selected two de novo KAND-associated mutations, V8M and Y89D, and performed all-atom MD simulations of WT or mutant motor domains interacting with the MT in their ATP-bound state (post-power stroke; PDB accession no. 4UXP; Atherton et al., 2014). Four replicate simulations of at least 200 ns each were performed, and analysis across replicate simulations was used to predict statistically significant (P < 10^−5^) differences in residue–residue distances between WT KIF1A and the KAND mutant motors.

**Table 2.** KAND-associated mutations that map to the KIF1A motor domain

KIF1A functional region	KAND-associated mutations
MT binding	α4: N272S, L278P
	Loop12: P305L
	α5: R307Q, R316W
	α6: R350G
Nucleotide binding and hydrolysis	P loop: T99M and G102S/D
	Loop 9 (switch 1): A202P, S215R, R216P/H/C, S217F
	Loop 11 (switch 2): L249Q, S252R, E253K, R254W/Q, A255V, T258M
Force generation and motor stepping	β1: V8M
	α1-β3: A85D, Y89D

**Figure 5.**
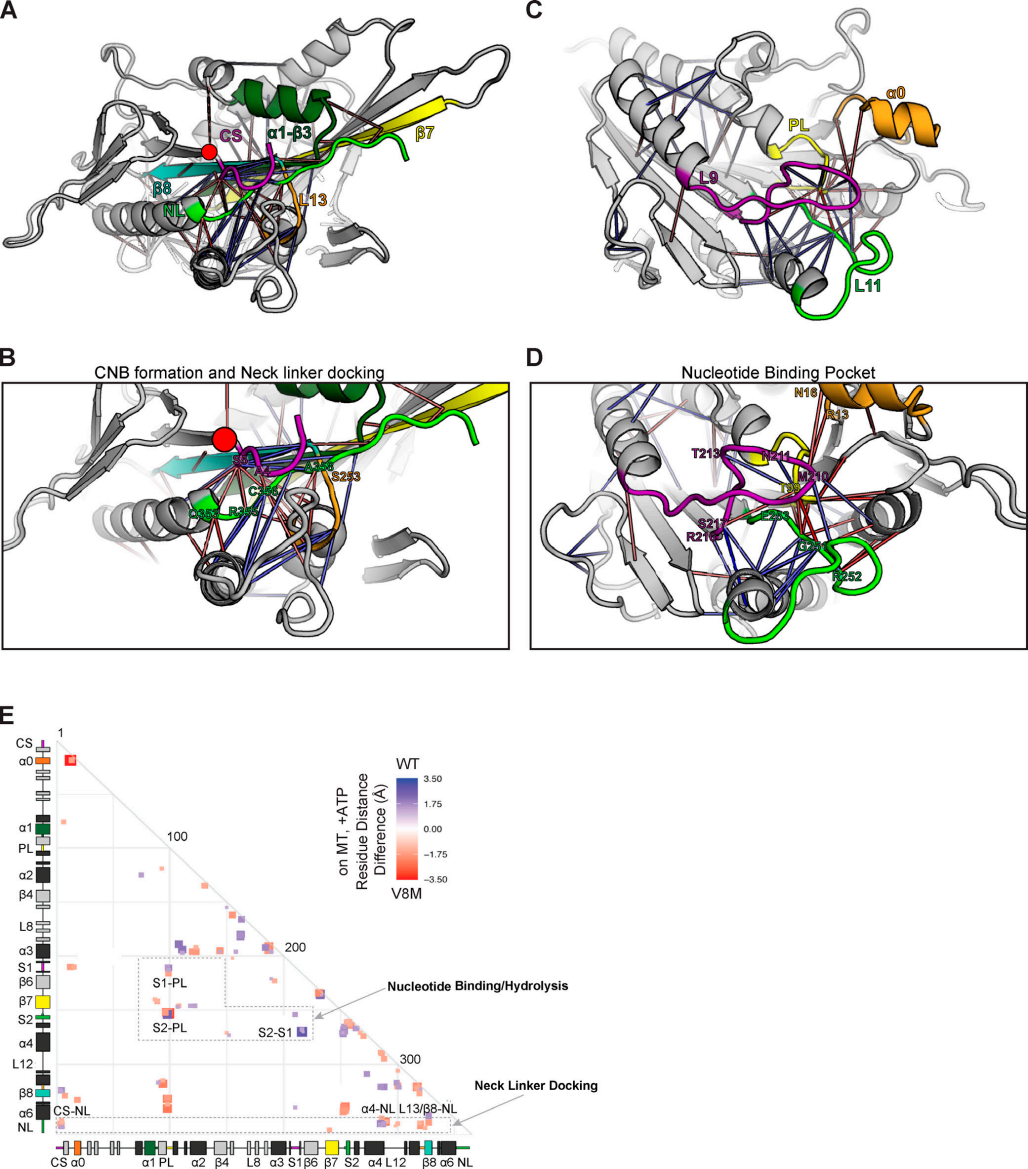
**MD simulations predict that the V8M mutation alters NL docking and catalytic site closure.** **(A–D)** Ribbon representation of the KIF1A motor domain in the ATP-bound, tubulin-bound state (PDB accession no. 4UXP). The V8M mutation (β1) is denoted as a red circle. Red lines depict residue–residue distances that are shorter in the V8M mutant, whereas blue lines depict residue–residue distances that are shorter in the WT motor. The magnitude of the distance change is indicated by line color intensity. **(A and B)** View of the NL docking pocket. In this post–power stroke state, the NL (green) is docked along the core motor domain. Secondary structures are indicated as purple, CS; dark green, α1-β3; yellow, β7; teal, β8; and orange, loop 13 (L13). **(C and D)** View of the nucleotide-binding pocket. Secondary structures are indicated as purple, Loop 9/switch1 (L9/S1); green, loop 11/switch2 (L11/S2); yellow, P loop (PL); and orange, α0. **(E)** Differences in residue–residue distances between WT KIF1A and V8M mutant motor in the ATP-bound, tubulin-bound state were determined in MD simulations. The secondary structure elements are laid out along the x and y axes with α-helices in black, β-strands in gray, or colored according to A. Residue–residue interactions that are significantly (P < 10^−5^) shorter in V8M mutant (red) or the WT motor (blue) are displayed on the grid. The magnitude of the distance change is indicated by color intensity.

For the V8M mutation, MD simulations predict local changes in residue–residue interactions important for NL-dependent motor stepping and force generation (Fig. 5, A, B, and E). Enhanced interactions are observed between the initial residues of β9 of the NL and the second residue (S6) of the CS (Fig. 5, A and B, orange connection lines; Fig. 5 E, red box labeled “CS-NL”), which may contribute to CNB formation and force output. However, reduced interactions are observed for the remainder of β9 and elements that position it for NL docking. In particular, reduced interactions are observed between β9 and residues of α4 that make up the docking pocket (Fig. 5, A and B, blue connection lines; and Fig. 5 E, blue box labeled “α4-NL”). Thus, the V8M mutation may position the CS such that it sterically occludes the NL’s access to the docking pocket. The MD simulations also predict reduced interactions between elements important for coordinating and hydrolyzing nucleotide (Fig. 5, C and D, blue connection lines; and Fig. 5 E, boxes labeled “S1-PL” and “S2-S1”), suggesting that the V8M mutant motor may have problems coordinating and/or hydrolyzing ATP and therefore have a reduced velocity compared with WT motors.

**Figure 6.**
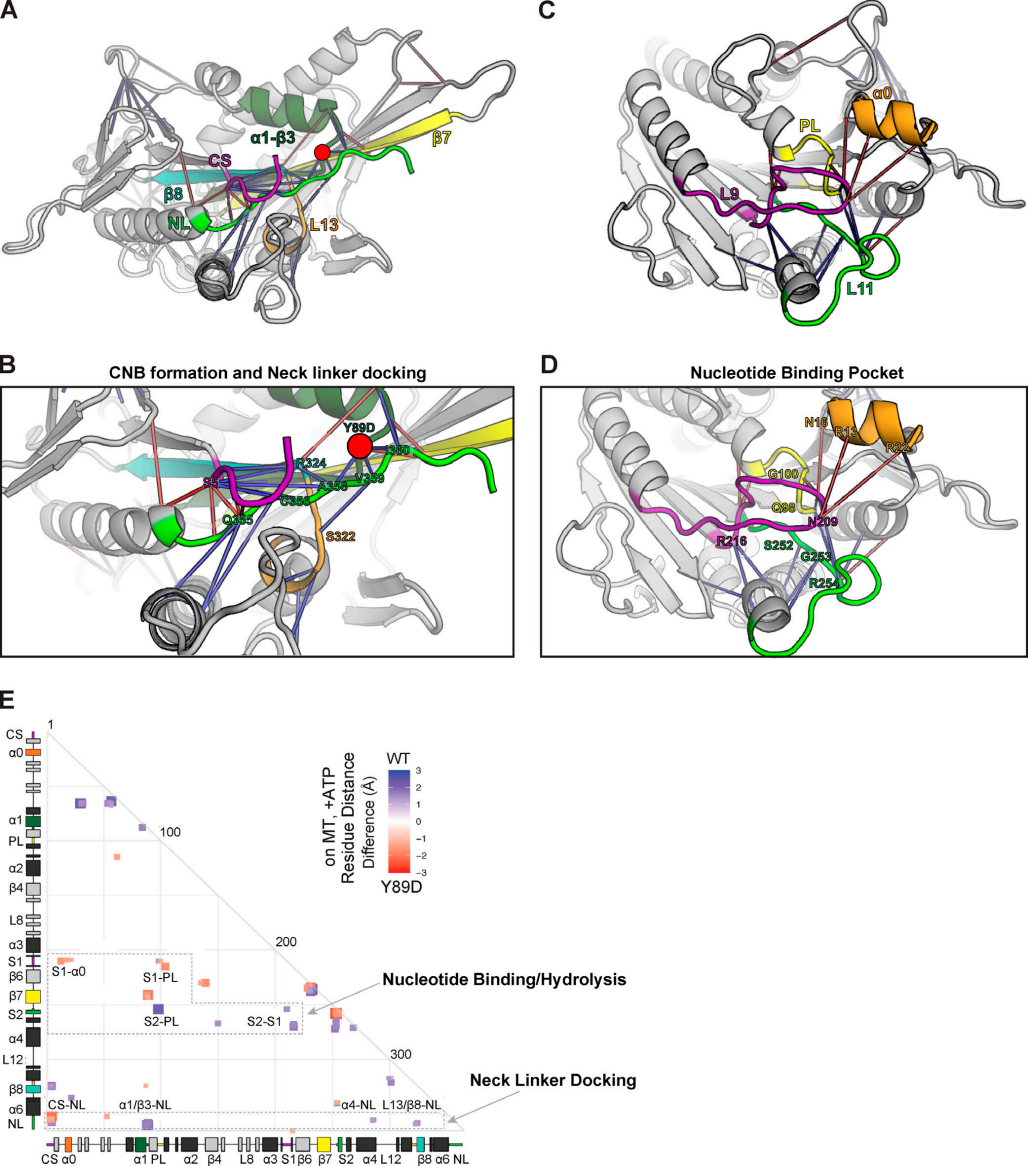
**MD simulations predict that the Y89D mutation alters NL docking and catalytic site closure.** **(A–D)** Ribbon representation of the KIF1A motor domain in the ATP-bound, tubulin-bound state (PDB accession no. 4UXP). The Y89D mutation (α1-β3) is denoted as a red circle. Red lines depict residue–residue distances that are shorter in the Y89D mutant, whereas blue lines depict residue–residue distances that are shorter in the WT motor. The magnitude of the distance change is indicated by line color intensity. **(A and B)** View of the NL docking pocket. In this post–power stroke state, the NL (green) is docked along the core motor domain. Secondary structures are indicated as purple, CS; dark green, α1-β3; yellow, β7; teal, β8; and orange, loop 13 (L13). **(C and D)** View of the nucleotide-binding pocket. Secondary structures are indicated as purple, loop 9/switch1 (L9/S1); green, loop 11/switch2 (L11/S2); yellow, P loop (PL); and orange, α0. **(E)** Differences in residue–residue distances between WT KIF1A and the Y89D mutant motor in the ATP-bound, tubulin-bound state determined in MD simulations. The secondary structure elements are laid out along the x and y axes with α-helices in black, β-strands in gray, or colored according to A. Residue–residue interactions that are significantly (P < 10^−5^) shorter in Y89D mutant (red) or the WT motor (blue) are displayed on the grid. The magnitude of the distance change is indicated by color intensity.

For the Y89D mutation, the MD simulations predict more severe restrictions on NL docking and thus a greater impact on force generation. Specifically, reduced interactions are observed for positioning β9 of the NL in the α4-lined docking pocket (Fig. 6, A and B, blue connection lines; and Fig. 6 E, blue box labeled “α4-NL”) and for subsequent docking of β10 along the core motor domain (Fig. 6, A and B, blue lines; and Fig. 6 E, blue boxes labeled “α1/β3-NL” and “L13/β8-NL”). In addition, the MD simulations revealed mixed effects between elements in the nucleotide-binding pocket. There are enhanced interactions between elements important for gating and capture of nucleotide (Fig. 6, C and D, orange connection lines; and Fig. 6 E, red boxes labeled “S1-α0”) as well as reduced interactions between elements important for nucleotide hydrolysis and exchange (Fig. 6, C and D, blue connection lines; and Fig. 6 E, blue boxes labeled “S2-PL” and “S2-S1”; Cao et al., 2014; Hwang et al., 2017; Kikkawa et al., 2001; Shang et al., 2014). Therefore, these results suggest that although the mutant motor may have no restrictions on binding ATP, it may display a reduced ability to hydrolyze ATP and undergo processive motility.

### V8M and Y89D mutations reduce force generation of KIF1A motors

**Figure 7.**
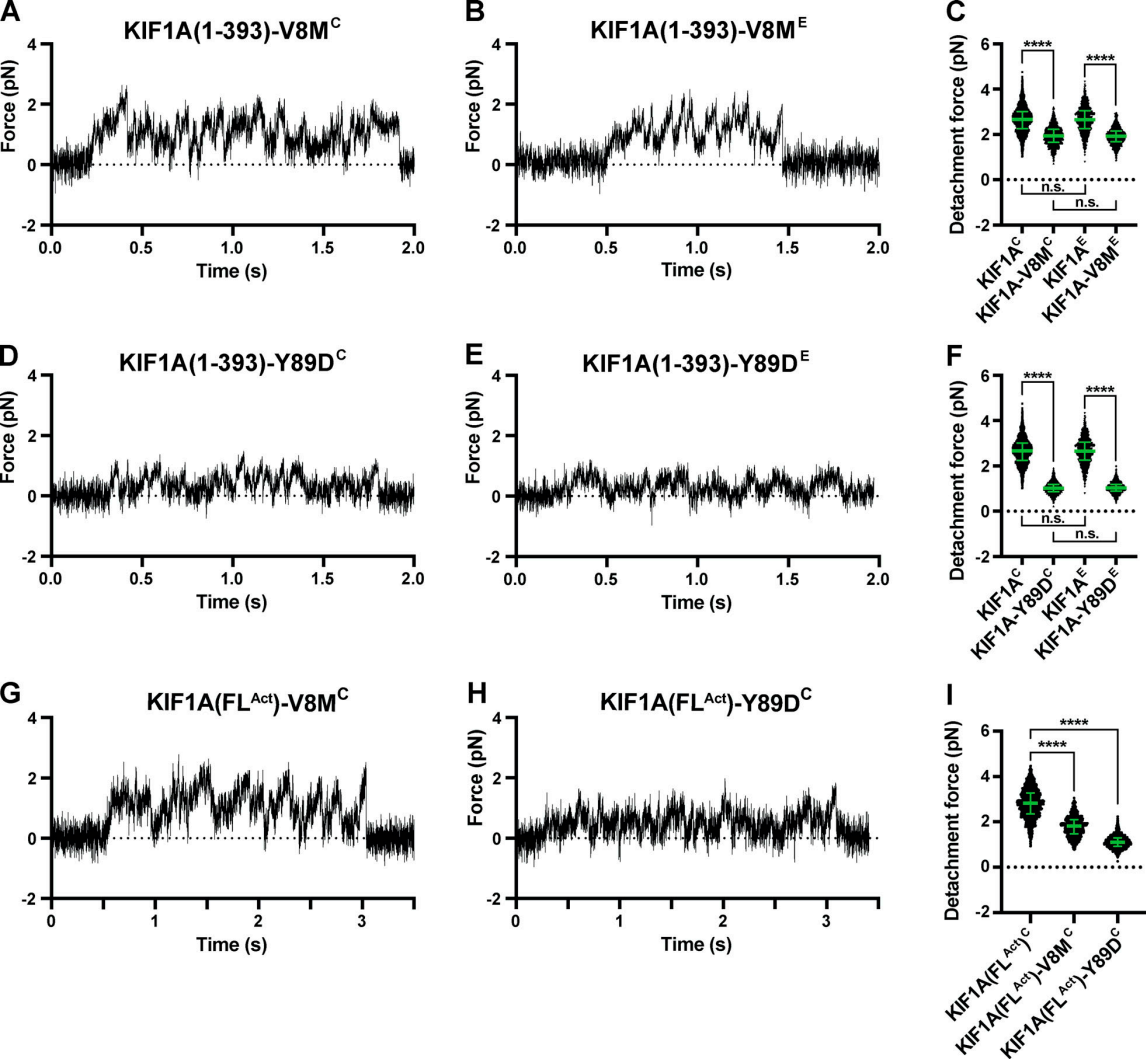
**V8M and Y89D mutations result in decreased force output for KIF1A motors.** **(A, B, D, and E)** Representative force versus time records of bead movement driven by single molecules of KIF1A(1–393)-V8M^C^ (A), KIF1A(1–393)-V8M^E^ (B), KIF1A(1–393)-Y89D^C^ (D), or KIF1A(1–393)-Y89D^E^ (E). *k* = 0.04–0.05 pN/nm. **(C)** Detachment forces of KIF1A(1–393)^C^ (2.66 [2.25, 3.01] pN, *n* = 1,912), KIF1A(1–393)-V8M^C^ (1.94 [1.65, 2.22] pN, *n* = 1,343], KIF1A(1–393)^E^ (2.65 [2.25, 3.05] pN, *n* = 1,044), and KIF1A(1–393)-V8M^E^ (1.92 [1.68, 2.15] pN, *n* = 1,032). Statistical significance was determined using an unpaired Welch’s *t* test (****, P < 0.0001). **(F)** As in C, but for KIF1A(1–393)-Y89D^C^ (1.02 [0.87, 1.19] pN, *n* = 1,468) and KIF1A(1–393)-Y89D^E^ (1.03 [0.89, 1.19] pN, *n* = 1,213). **(G and H)** As in A, but for KIF1A(FL^Act^)-V8M^C^ (G) and KIF1A(FL^Act^)-Y89D^C ^(H). *k* = 0.04–0.05 pN/nm. **(I)** As in C, but for KIF1A(FL^Act^)^C^ (2.82 [2.35, 3.27] pN, *n* = 1,433), KIF1A(FL^Act^)-V8M^C^ (1.81 [1.48, 2.10] pN, *n* = 1,024) and KIF1A(FL^Act^)-Y89D^C^ (1.10 [0.92, 1.29] pN, *n* = 1,022). Data were analyzed across two or three independent experiments. Green bars in C, F, and I indicate the median values with quartiles. n.s., not significant.

To examine the effects of the V8M and Y89D mutations on the force output of the motors, we again used optical tweezers and motors attached to beads under single-molecule conditions. We examined biotinylated KIF1A(1–393) motors containing the V8M or Y89D mutations in COS-7 cell lysates (KIF1A(1–393)-V8M/Y89D^C^) and GFP-tagged motors purified from *E. coli* bacteria (KIF1A(1–393)-V8M/Y89D)^E^). Regardless of expression system, the V8M and Y89D mutant motors were sensitive to small opposing forces exerted by the trap (Fig. 7, A–F). Both mutant motors displayed an impaired force output, as their detachment forces (2.0 [1.7, 2.2] pN and 1.0 [0.9, 1.2] pN (median [quartiles]) in COS-7 lysates; 1.9 [1.7, 2.2] pN and 1.0 [0.9, 1.2] pN purified from *E. coli* cells, respectively) were significantly reduced as compared with the WT motors (P < 0.0001 for KIF1A(1–393)-V8M^E^ and KIF1A(1–393)-Y89D^E^ compared with WT KIF1A(1–393)^E^, and for KIF1A(1–393)-V8M^C^ and KIF1A(1–393)-Y89D^C^ compared with WT KIF1A(1–393)^C^; Welch’s *t* test; Table 1). The reduced force output of the mutant motors is consistent with MD simulations that predict impaired docking of β9 and/or β10 of the NL to the core motor domain (Figs. 5 and 6). Interestingly, similar to the WT motor, the mutant motors detached from the MT before reaching a stall plateau and quickly rebounded to the MT after detaching (Fig. 7, A, B, D, and E), resulting in a clustering of force-generating events (Fig. 3 B).

We also tested the effects of the V8M and Y89D mutations on force generation in the context of the active, biotinylated FL motor (KIF1A(FL^Act^)-V8M/Y89D^C^). Similar to what we observed for the minimal dimeric motors, the detachment forces of the FL mutant motors were reduced compared with the WT protein with detachment forces of 1.8 (1.5, 2.1) pN (median [quartiles]) for KIF1A(FL^Act^)-V8M^C^ (Fig. 7, G and I) and 1.1 (0.9, 1.3) pN for KIF1A(FL^Act^)-Y89D^C^ (Fig. 7, H and I), as compared with the 2.8 (2.4, 3.3) pN of the WT FL motor (Table 1). These results indicate that NL docking is a critical factor for force generation by KIF1A motors. These results also demonstrate that KAND mutations V8M and Y89D result in impaired force generation for KIF1A motors.

### V8M and Y89D mutations relieve autoinhibition but active motors display impaired motility properties

**Figure 8.**
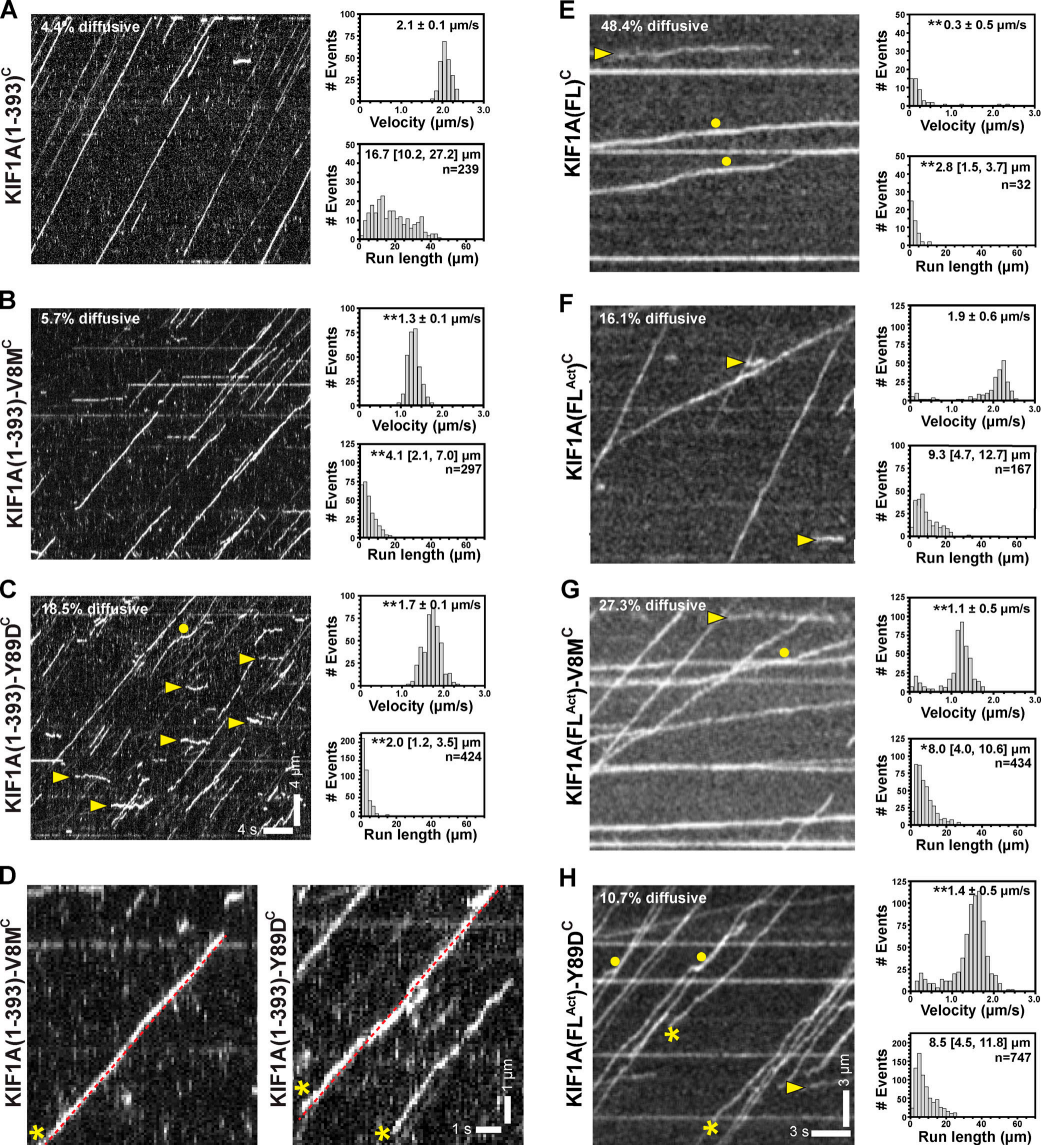
**V8M and Y89D mutations relieve autoinhibition of FL motors but reduce speed and processivity.** **(A–C)** Representative kymographs of single-molecule motility assays of KIF1A(1–393)^C^ (A), KIF1A(1–393)-V8M^C^ (B), and KIF1A(1–393)-Y89D^C^ (C) with time displayed on the x axis (scale bar = 4 s) and distance displayed on the y axis (scale bar = 4 µm). Yellow arrowheads, diffusive events; yellow circles, pauses. The processive motility events were quantified, and the data were plotted as histograms of single-motor velocities and run lengths. Velocities: mean ± SEM; **, P < 0.001 as compared with the WT motor (Student’s *t* test). Run lengths: median (quartiles); **, P < 0.001 as compared with the WT motor (Kruskal-Wallis test). KIF1A(1–393)^C^: *n* = 239 (4.4% diffusive); KIF1A(1–393)-V8M^C^: *n* = 297 (5.7% diffusive); KIF1A(1–393)-Y89D^C^: *n* = 424 (18.5% diffusive) across three independent experiments. **(D)** Magnified views of kymographs for KIF1A(1–393)-V8M^C^ (left) and KIF1A(1–393)-Y89D^C^ (right). Yellow asterisks, “wobbly” events that deviate from linear motility (dotted red line). **(E–H)** Representative kymographs from single-molecule motility assays of KIF1A(FL)^C^ (E), KIF1A(FL^Act^)^C^ (F), KIF1A(FL^Act^)-V8M^C^ (G), and KIF1A(FL^Act^)-Y89D^C^ (H) with time displayed on the x axis (scale bar = 3 s) and distance displayed on the y axis (scale bar = 3 µm). Yellow arrowheads, diffusive events; yellow asterisks, “wobbly” events that deviate from linear motility; yellow circles, pauses. The processive motility events were quantified, and the data were plotted as histograms of single-motor velocities and run lengths. Velocities: mean ± SEM; **, P < 0.001 as compared with the FL^Act^ motor (Student’s *t* test). Run lengths: median (quartiles); **, P < 0.001; *, P = 0.027 as compared with the FL^Act^ motor (Kruskal-Wallis test). KIF1A(FL)^C^: *n* = 62 (48.4% diffusive); KIF1A(FL^Act^)^C^: *n* = 199 (16.1% diffusive); KIF1A(FL^Act^)-V8M^C^: *n* = 434 (27.3% diffusive); KIF1A(FL^Act^)-Y89D^C^: *n* = 747 (10.7% diffusive) across three independent experiments.

We next used fluorescence-based single-molecule motility assays to examine the behavior of WT or KAND mutant KIF1A motors under unloaded conditions. Minimal dimeric WT KIF1A(1–393)^C^ motors tagged with monomeric NeonGreen (mNG) displayed fast (2.1 ± 0.1 µm/s [mean ± SEM]) and superprocessive (16.7 [10.2, 27.2] µm; median [quartiles]) motility with a high landing rate of 10.7 ± 0.6 events⋅min^−1^⋅nM^−1^⋅µm^−1^ (mean ± SEM; Fig. 8 A and Table 3), consistent with previous work (Soppina et al., 2014).

**Table 3.** Unloaded single-molecule motility properties

KIF1A motor	Velocity (μm/s)^a^	Run length (μm)^b^	Landing rate (events⋅min^−1^⋅nM^−1^⋅μm^−1^)^c^
KIF1A(1–393)^C^	2.1 ± 0.1	16.7 (10.2, 27.2)	10.7 ± 0.6
KIF1A(1–393)-V8M^C^	1.3 ± 0.1^e^	4.1 (2.1, 7.0)^f^	1.5 ± 0.1^e^
KIF1A(1–393)-Y89D^C^	1.7 ± 0.1^e^	2.0 (1.2, 3.5)^f^	2.8 ± 0.1^e^
KIF1A(1–393)-WT^E^	2.5 ± 0.2	12.2 (6.7, 18.4)	ND
KIF1A(1–393)-V8M^E^	1.3 ± 0.1	7.3 (4.4, 12.2)	ND
KIF1A(1–393)-Y89D^E^	1.7 ± 0.2	6.3 (4.0, 10.7)	ND
KIF1A(FL)^C^	0.3 ± 0.1	2.8 (1.5, 3.7)	2.0 ± 0.2
KIF1A(FL^Act^)^C^	1.9 ± 0.1	9.3 (4.7, 12.7)	2.8 ± 0.6
KIF1A(FL^Act^)-V8M^C^	1.1 ± 0.1^d^	8.0 (4.0, 10.6)^g^	5.1 ± 0.4^d^
KIF1A(FL^Act^)-Y89D^C^	1.4 ± 0.1^d^	8.5 (4.5, 11.8)^h^	5.9 ± 0.4^d^

a Data are reported as mean ± SEM.

b Data are reported as median (quartiles).

c Includes diffusive + processive events.

d P < 0.001 (Student’s *t* test) compared with KIF1A(FL^Act^)^C^.

e P < 0.001 (Student’s *t* test) compared with KIF1A(1-393)^C^.

f P < 0.001 (Kruskal-Wallis test) compared with KIF1A(1-393)^C^.

g P = 0.027 (Kruskal-Wallis test) compared with KIF1A(FL^Act^)^C^.

h P = 0.38 (Kruskal-Wallis test) compared with KIF1A(FL^Act^)^C^.

KIF1A(1–393)-V8M^C^ mutant motors displayed significant decreases in overall velocity (1.3 ± 0.1 µm/s), processivity (4.1 [2.1, 7.0] µm), and landing rate (1.5 ± 0.1 events⋅min^−1^⋅nM^−1^⋅µm^−1^; Fig. 8 B and Table 3). Given the measured step size of ~8 nm (Fig. 1 H), the resulting stepping rates (~263/s for WT KIF1A and 163/s for V8M) suggest an ~40% reduction of the apparent ATPase rate for KIF1A(1–393)-V8M^C^. This analysis assumes that the WT and mutant motors take forward steps with a similar probability and efficiency. In support of this assumption, even under 1–2 pN loads, KIF1A(1–393)^C^ and KIF1A(1–393)-V8M^C^ show the same stepping behavior (P < 0.51, Welch’s *t* test) and take forward steps with a probability of ~98% (Fig. S3 I). Given the recent finding that ATP hydrolysis triggers forward stepping of KIF1A (Zaniewski et al., 2020), these analyses are consistent with the MD simulations that predict allosteric effects on reduced catalytic site closure and reduced ATP hydrolysis (Fig. 5).

KIF1A(1–393)-Y89D^C^ mutant motors also displayed significant decreases in velocity (1.7 ± 0.1 µm/s), processivity (2.0 [1.2, 3.5] µm), and landing rate (2.8 ± 0.1 events⋅min^−1^⋅nM^−1^⋅µm^−1^; Fig. 8 C and Table 3). Further examination of the kymographs indicated two additional differences in the motility behavior of Y89D mutant motors. First, the tracks of Y89D motility were not smooth; rather, the motors appeared to “wobble” as they walked along the MT track (Fig. 8 D). Second, a large number of nonproductive, diffusive events (net displacement along the MT <200 nm) were observed (Fig. 8 C, yellow arrowheads). The Y89D mutant motors displayed a greater percentage of diffusive events (18.4% of events; Fig. 8 C) than the WT (4.2%; Fig. 8 A) or V8M motors (5.7%; Fig. 8 B). The increase in diffusive events for the Y89D mutant motors suggests that the motor often engages in a weak MT-binding state.

To ensure that the minimal dimeric KIF1A(1–393) motors reflect the behavior of the FL motor, we performed similar single-molecule motility assays with FL KIF1A in COS-7 cell lysates (KIF1A(FL)^C^). Consistent with KIF1A existing in an autoinhibited state (Hammond et al., 2009; Okada et al., 1995; Soppina et al., 2014), very few KIF1A(FL)^C^ motility events were observed, and, of those, nearly half (48.4% of all events) were diffusive events (Fig. 8 E). When motile, the autoinhibited FL motor moved slowly (0.3 ± 0.1 µm/s) and with relatively low processivity (2.8 [1.5, 3.7] µm; Fig. 8 E and Table 3). In contrast, the active KIF1A(FL^Act^)^C^ motor displayed a dramatic increase in the number of events, with nearly all events (83.9% of all events) involving processive motility (Fig. 8 F and Table 3). The KIF1A(FL^Act^)^C^ motors also moved with a high speed (1.9 ± 0.1 µm/s) and run length (9.3 [4.7, 12.5] µm; Fig. 8 F and Table 3), similar to the minimal dimeric KIF1A(1–393) motor (Table 3) and consistent with previous work (Huo et al., 2012).

FL motors containing the V8M or Y89D mutations also displayed a dramatic increase in landing events, suggesting that both disease mutations relieve autoinhibition. While these results agree with previous work demonstrating that the V8M mutation relieves the autoinhibition of FL WT KIF1A (Chiba et al., 2019), we were surprised that our V8M and Y89M mutant motors displayed significantly higher landing rates (5.1 ± 0.4 events⋅min^−1^⋅nM^−1^⋅µm^−1^ and 5.9 ± 0.4 events⋅min^−1^⋅nM^−1^⋅µm^−1^, respectively) than the previously demonstrated active version, KIF1A(FL^Act^) (Fig. 8, G and H; and Table 3). These results suggest that the V483N mutation in the first coiled-coil domain of KIF1A only partially relieves autoinhibition and that both KAND-associated mutations in the motor domain are more potent at preventing the autoinhibitory state of KIF1A. When processive, both the KIF1A(FL^Act^)-V8M^C^ and KIF1A(FL^Act^)-Y89D^C^ mutant motors moved with significantly slower speeds (1.1 ± 0.1 µm/s and 1.4 ± 0.1 µm/s, respectively; Fig. 8, G and H; and Table 3) than KIF1A(FL^Act^). The mutant motors also underwent shorter runs of 8.0 (4.0, 10.6) µm and 8.5 (4.5, 11.8) µm, respectively, than KIF1A(FL^Act^) (Fig. 8, G and H; and Table 3). The KIF1A(FL^Act^)-Y89D^C^ mutant motor often appeared to “wobble” or undergo rapid changes in speed and/or direction as it walked (Fig. 8 H), whereas the V8M mutant motor engaged in more diffusive events (27.2%; Fig. 8 G) than KIF1A(FL^Act^) (16.1%; Fig. 8 F) or Y89D (10.7%; Fig. 8 H) motors (Table 3).

Finally, to ensure that the changes in motility of the V8M and Y89D motors were due to direct effects on motor behavior rather than to indirect alterations in the cell lysate context, we purified KIF1A(1–393) WT, V8M, and Y89D motors from *E. coli*. The KIF1A(1–393)^E^ motors displayed fast (2.5 ± 0.2 µm/s, mean ± SEM; Fig. S1, D–F) and superprocessive (12.2 [6.7,18.4] µm; Fig. S1 F) motility. Similar to the mammalian-expressed mutant motors, the recombinant KIF1A(1–393)-V8M^E^ and KIF1A(1–393)-Y89D^E^ mutant motors were slower (1.3 ± 0.1 µm/s and 1.7 ± 0.2 µm/s, respectively; Fig. S1, G, H, J, and K) than the WT motor (Table 3). The mutant motors also displayed a reduced processivity (7.3 [4.4,12.2] µm and 6.3 [4.0, 10.7] µm, respectively; Fig. S1, I and L) as compared with the KIF1A(1–393)^E^ motor (Table 3). Overall, we conclude that, as homodimeric motors, the V8M and Y89D mutations result in impairments to both the velocity and processivity of KIF1A.

### V8M and Y89D mutations reduce velocity and processivity of heterodimeric motors

**Figure 9.**
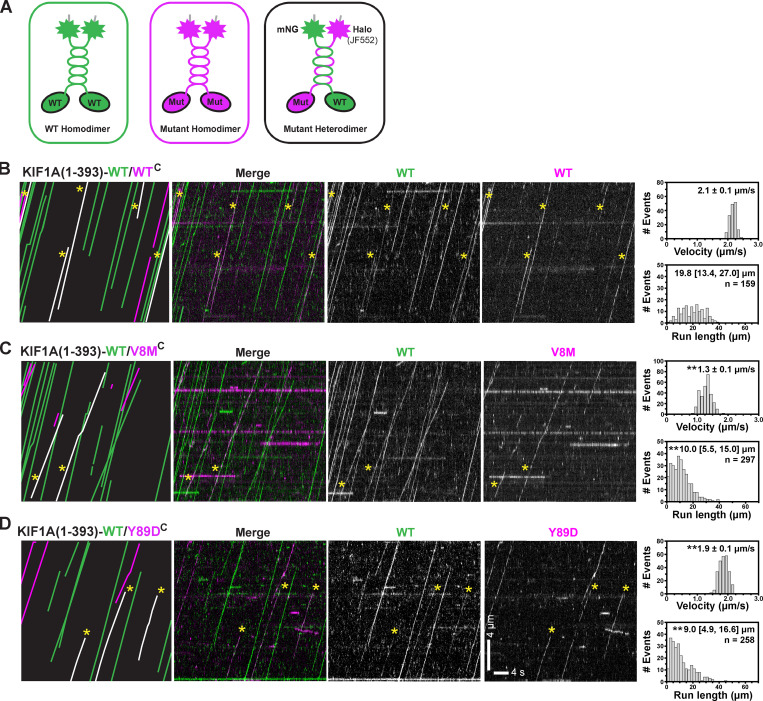
**Heterodimeric WT/V8M and WT/Y89D mutant motors display decreased processivity.** **(A)** Schematic of strategy for generating heterodimeric WT/mutant KIF1A(1–393) motors. WT motors tagged with mNG were coexpressed with V8M or Y89D motors tagged with Halo-FLAG and labeled with JF552, thus generating three populations of motors: homodimeric WT motors (green), homodimeric mutant motors (magenta), and heterodimeric WT/mutant motors (green + magenta). **(B–D)** Single-molecule motility assays of KIF1A(1–393)-WT/WT^C^ (B), KIF1A(1–393)-WT/V8M^C^ (C), or KIF1A(1–393)-WT/Y89D^C^ (D). Representative kymographs are shown with time displayed on the x axis (scale bar, 4 s) and distance displayed on the -y axis (scale bar, 4 µm). Motility events of heterodimeric motors are indicated by yellow asterisks across images and by white lines in cartoon kymographs (far left). The processive motility events were quantified, and the data were plotted as histograms for single-motor velocities and run lengths. Velocities: mean ± SEM; **, P < 0.001 as compared with the WT/WT motor (Student’s *t* test). Run lengths: median (quartiles); **, P < 0.001 as compared with the WT/WT motor (Kruskal-Wallis test). KIF1A(1–393)-WT/WT^C^, *n* = 159; KIF1A(1–393)-WT/V8M^C^, *n* = 297; KIF1A(1–393)-WT/Y89D^C^, *n* = 258 events across three independent experiments.

**Figure S5.**
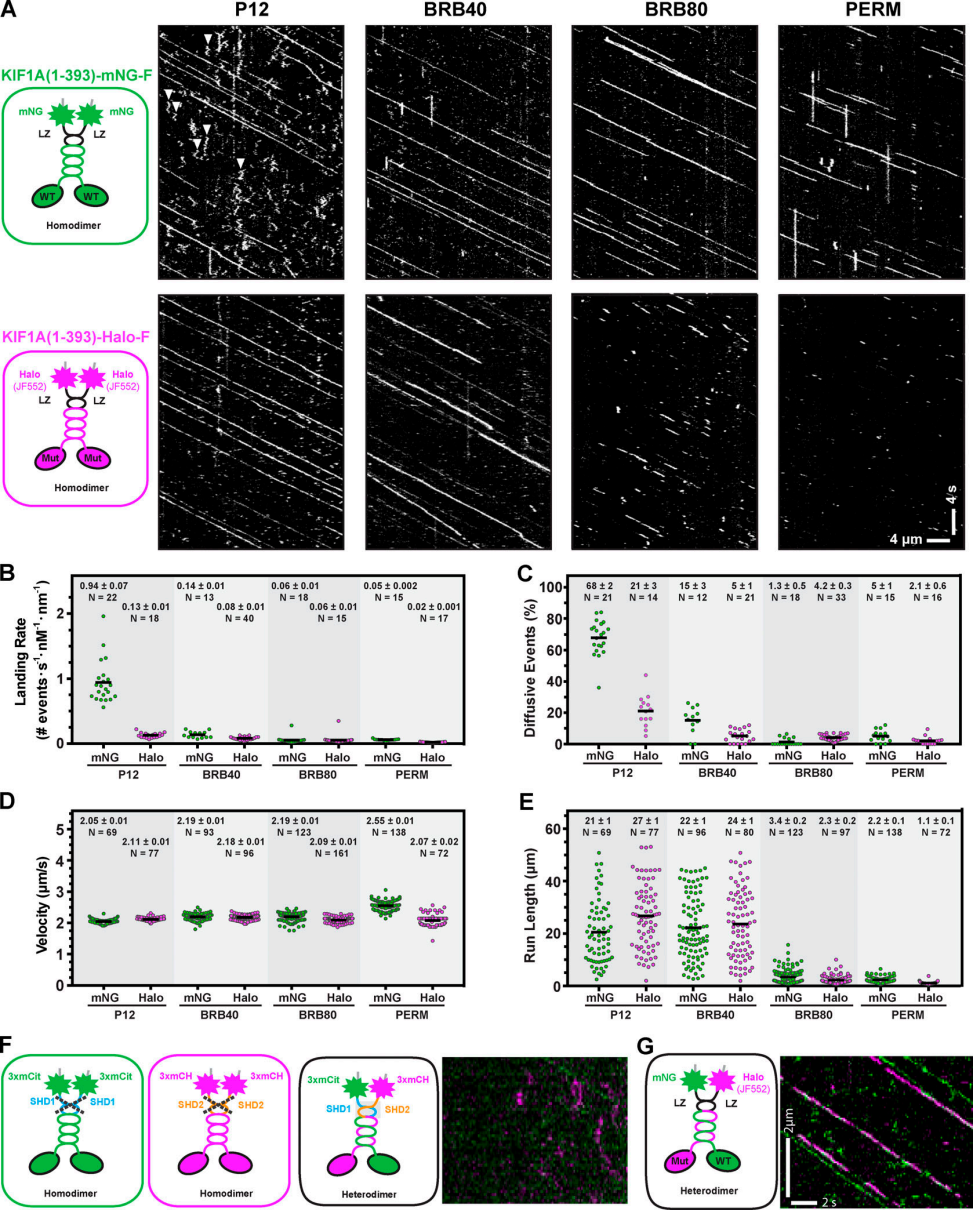
**Influence of fluorescence tag and buffer conditions on KIF1A motility and strategies for designing heterodimeric motors.** **(A)** Motility properties of KIF1A motors tagged with mNG-Flag or Halo-FLAG/JF552. Motors in COS-7 lysates were analyzed in standard single-molecule motility assays using TIRF microscopy. Representative kymographs are shown with time displayed on the y axis (scale bar, 4 s) and distance displayed on the x axis (scale bar, 4 µm). White arrowheads indicate motility events scored as diffusive. **(B–E)** Quantification of motility properties. The kymographs were used to determine landing rates (diffusive and processive events with dwell times >400 ms, each spot indicates the events on a single MT; B), frequency of diffusive events (net displacement <200 nm (each spot indicates the events on a single MT; C), velocity (each dot represents a single motor; D), and run length (each dot represents a single motor; E). Consistent with previous studies (Norris et al., 2015), buffer conditions had little effect on velocity but did affect the other parameters in a tag-dependent manner. **(F and G)** Strategies to generate heterodimeric motors. F shows that synthetic heterodimerization (SHD) sequences SHD1 and SHD2 do not homodimerize (left and middle) but rather result in heterodimer formation (right; Albracht et al., 2014). KIF1A(1–393)-SHD1 was tagged with three tandem monomeric citrine fluorescent proteins (green), and KIF1A(1–393)-SHD2 was tagged with three tandem monomeric Cherry proteins (magenta). Coiled-coil prediction software (Delorenzi and Speed, 2002) was used to ensure that the SHD sequences were placed in register with the native KIF1A neck coil. G is the same as in F, but the GCN4 LZ sequence was used to maintain the dimer state. To test for heterodimer formation, lysates were prepared from COS-7 cells cotransfected with plasmids coding for KIF1A(393)-SHD1-3xmCit and KIF1A(393)-SHD2-3xmCH motors (F) or KIF1A(393)-LZ-mNG and KIF1A(393)-LZ-Halo/JF552 motors (G). Kymographs were generated from single-molecule assays [right panels; time is displayed on the y axis (scale bar, 2 s), and distance is displayed on the x axis (scale bar, 2 µm)]. Very few heterodimeric (magenta/green) spots were detected for motors tagged with the SHD sequences, whereas fast, superprocessive motility was observed for the LZ-stabilized dimeric KIF1A motors.

The V8M and Y89D mutations found in KAND patients are inherited in an autosomal dominant manner, indicating that the disease allele can influence transport even in the presence of a WT allele. We thus examined the effect of the KAND mutations in the heterodimeric state where one motor domain is WT and the second motor domain harbors a KAND mutation. We tested several strategies for generating heterodimeric motors but were unable to achieve complete heterodimer formation (Fig. S5 F). We thus cotransfected COS-7 cells with plasmids for expression of WT motors tagged with mNG and KAND mutant motors tagged with Halo and FLAG tags (Fig. 9 A) and tested several imaging conditions to avoid artifacts related to either of the tags (Fig. S5, A–E). From the kymographs, motility events of heterodimeric motors were scored as comotility in both the mNG and Halo(JF552) fluorescence channels (Fig. 9, B–D).

KIF1A(1–393)-WT/WT^C^ motors tagged with both mNG and Halo(JF552) fluorophores displayed fast velocities (2.1 ± 0.1 µm/s) and long run length (19.8 [13.4, 27.0] µm; Fig. 9 A) comparable to the homodimeric motors studied above. The presence of the V8M motor domain resulted in a significant (P < 0.001, Student’s *t* test) reduction in velocity (1.3 ± 0.1 µm/s; Fig. 9 B) such that the heterodimeric KIF1A(1–393)-WT/V8M^C^ motor’s velocity is comparable to that of homodimeric V8M/V8M^C^ mutant motors. In addition, the processivity of KIF1A(1–393)-WT/V8M^C^ motors (10.0 [5.5, 15.0] µm; Fig. 9 B) was significantly (P < 0.001, Kruskal-Wallis test) reduced compared with WT/WT^C^ motors but was not as severely hindered as in the V8M/V8M^C^ motors.

The presence of the Y89D motor domain had minimal effects on velocity in the context of the heterodimeric KIF1A(1–393)-WT/Y89D^C^ motor (1.9 ± 0.1 µm/s; Fig. 9 D) as compared with the WT/WT^C^ motor but resulted in a significant (P < 0.001, Kruskal-Wallis test) reduction in processivity (9.0 [4.9, 16.6] µm; Fig. 9 C), although these effects were not as severe as observed for the Y89D/Y89D homodimeric motors. In addition, the KIF1A(1–393)-WT/Y89D^C^ heterodimeric motors did not exhibit the diffusive behavior of the KIF1A(1–393)-Y89D/Y89D^C^ homodimeric motors. Collectively, these results suggest that, when paired with a WT motor domain in a heterodimeric motor, both the V8M and Y89D mutations cripple the overall motility with greater effects on motor processivity than motor speed.

### Organelle transport driven by mutant motors in cells is delayed

**Figure 10.**
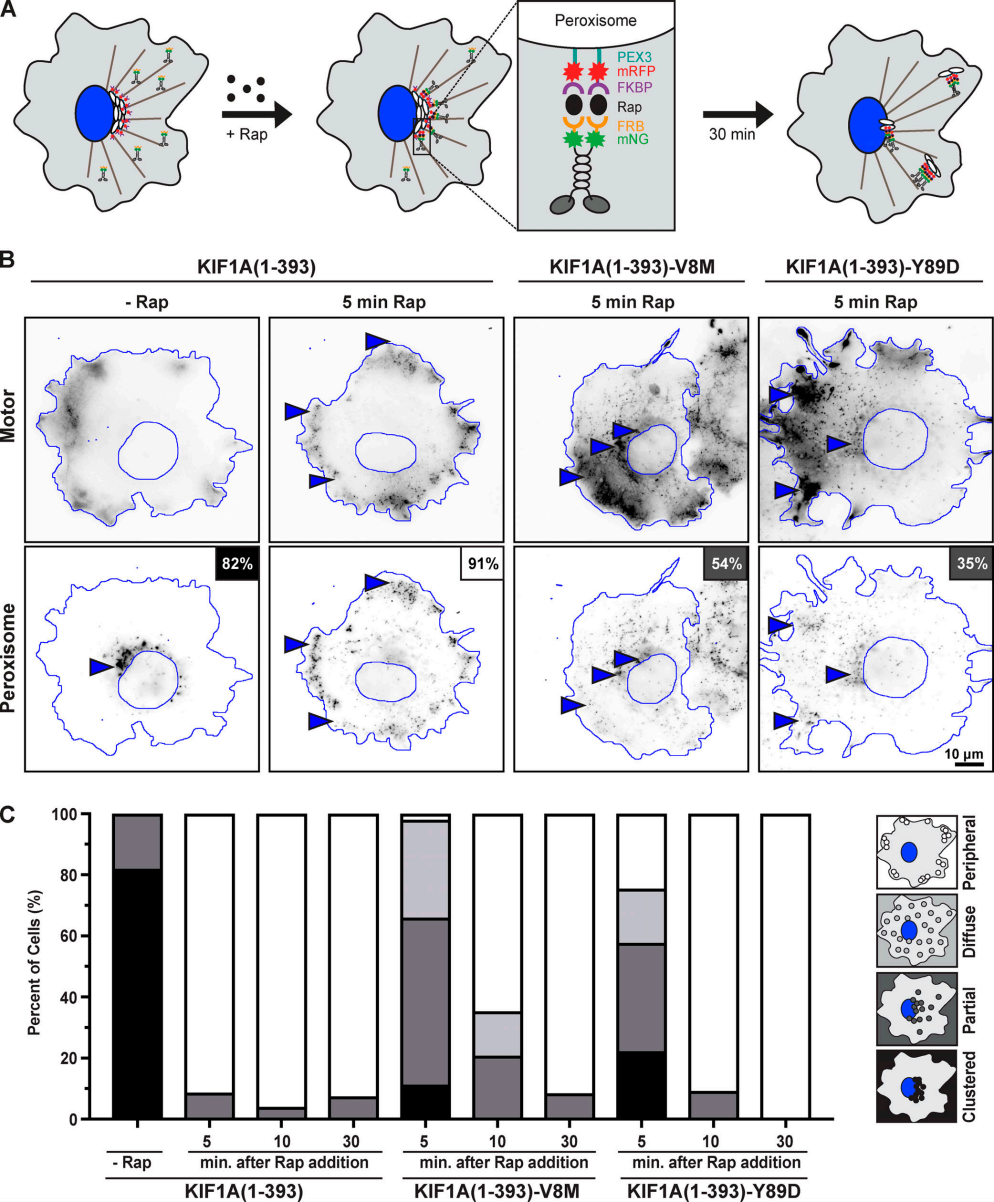
**V8M and Y89D mutant motors show delayed transport of membrane-bound cargo in cells.** **(A)** Schematic of the peroxisome dispersion assay. A kinesin motor fused to monomeric mNG and an FRB domain (KIF1A(1–393)-mNG-FRB) is coexpressed in COS-7 cells with a peroxisome-targeting sequence (PEX3) fused to mRFP and an FKBP domain (PEX3-mRFP-FKBP). Addition of rapamycin (+Rap) causes heterodimerization of the FRB and FKBP domains and recruitment of motors to the peroxisome membrane. Recruitment of active motors drives cargo dispersion to the cell periphery. **(B)** Representative images of peroxisome dispersion before (−Rap) and 10 min after (10 min Rap) recruitment of WT or mutant motors to the peroxisome surface. Blue lines indicate the nucleus and periphery of each cell. Blue arrowheads indicate peroxisomes. Scale bar, 10 µm. Percentages in the upper right corner indicate the percentage of cells with the indicated dispersion phenotype: black, clustered peroxisomes; dark gray, partially dispersed peroxisomes; light gray, diffusely dispersed peroxisomes; white, peripherally dispersed peroxisomes. **(C)** Qualitative analysis of peroxisome dispersion. Cells were scored as clustered (black), partially dispersed (dark gray), diffusely dispersed (light gray), or peripherally dispersed (white). The phenotypes of *n* ≥ 43 cells across three experiments were combined into a stacked bar plot for each construct at each time point.

We hypothesized that the reduced force output and motility properties of the KAND mutant motors would impair their ability to drive cargo transport in cells. However, it remained possible that multiple motors could compensate when working in teams. To examine the ability of WT and KAND mutant motors to work in a team to drive cargo transport in cells, we used the peroxisome dispersion assay (Budaitis et al., 2019; Efremov et al., 2014; Kapitein et al., 2010; Schimert et al., 2019) to recruit multiple motors to the surface of peroxisomes and monitored their ability to drive organelle transport to the cell periphery (Fig. 10 A). Peroxisome location before and after motor recruitment was qualitatively scored as clustered (black), partially dispersed (dark gray), diffusively dispersed (light gray), or peripherally dispersed (white; Fig. 10 C).

COS-7 cells were cotransfected with a plasmid for the expression of WT or KAND mutant KIF1A(1–393) motors tagged with mNG and FRB domain and a plasmid for the expression of a peroxisome-targeted PEX–monomeric RFP (mRFP)–FKBP fusion protein. In the absence of rapamycin, the PEX-mRFP-FKBP peroxisomes were largely clustered in the center of the cell (93% of cells had clustered peroxisomes; Fig. 10, B and C), whereas KIF1A(1–393)-mNG-FRB motors accumulated at the periphery of the cell (Fig. 10 B). Addition of rapamycin resulted in recruitment of motors to the peroxisome surface via dimerization of the FRB and FKBP domains, and motor activity drove dispersion of peroxisomes to the cell periphery. 5 min after recruitment of WT motors, 91% of cells (42/46) had peroxisomes dispersed to the periphery of the cell (Fig. 10, B and C). In contrast, 5 min after recruitment of teams of V8M or Y89D mutant motors, the peroxisomes failed to reach the periphery of the cell. Rather, 54% (29/53) of V8M-expressing cells and 35% (16/45) of Y89D-expressing cells displayed only partial peroxisome dispersion (Fig. 10, B and C).

We hypothesized that the impaired motility and force generation properties of the V8M and Y89D motors could be overcome if the motors were given more time to complete the transport event. We thus repeated the peroxisome dispersion assay but waited 10 or 30 min after recruitment of teams of V8M or Y89D mutant motors to assess peroxisome localization. At 10 min after rapamycin-induced motor recruitment, 65% (31/48) of cells expressing the V8M mutant motor and 91% (39/43) of cells expressing the Y89D mutant motor displayed peripheral dispersion of the peroxisomes as compared with 96% (47/49) of cells expressing the WT motor (Fig. 10 C). After 30 min of motor recruitment, the V8M and Y89D mutant motors were able to achieve peroxisome dispersion (91% [43/47] and 100% [49/49] of cells, respectively) to the same extent as the WT motor (92% [49/53] of cells; Fig. 10 C). Collectively, these results suggest that the reduced force output, processivity, and velocity of the V8M and Y89D mutant motors result in impaired cargo transport in cells.

## Discussion

Kinesin-3 motors drive a large number of intracellular trafficking events; yet, their ability to generate and sustain force is largely untested. We find that, unlike conventional kinesin-1, mammalian KIF1A motors and *C. elegans* UNC-104 motors detach from the MT track under low forces. Furthermore, both motors rapidly reattach to the MT and continue forward motion, a property that may enable fast transport of presynaptic vesicles over long distances. We find that the disease-associated V8M and Y89D mutations compromise the force output of single motors and result in decreased velocity, processivity, and landing rate via allosteric effects on regions of the core motor domain responsible for NL docking and the coordination and binding of nucleotide. The mutant motors also show a delay in their ability to transport cargo in cells. These results highlight the role of the NL in force generation for kinesin motors.

### KIF1A readily detaches from MTs under load but rapidly reattaches for persistent motility

Previous studies of kinesin-3 motors focused on their striking motility properties under no-load conditions. Here, we analyze two members of the kinesin-3 family under load and note several interesting aspects of KIF1A force generation that are likely to impact its cellular functions.

First, KIF1A and UNC-104 motors have a high force-dependent detachment rate. While individual KIF1A motors can move against a resisting force to the point of movement cessation (stall force), they more often detach from the MT track. This behavior may suggest that KIF1A is sensitive to vertical force components (forces normal to the MT surface), which increase as the angle between the bead-bound motor and the MT surface increases. Consistent with this idea, we find that the longer FL KIF1A motor, which can adopt a smaller angle with the surface, stalls for a longer time than the shorter tail-truncated KIF1A. While such a behavior has also been reported for kinesin-1 (Khataee and Howard, 2019; Pyrpassopoulos et al., 2020), KIF1A appears to be more sensitive to vertical forces than single kinesin-1 motors, which appear to resist detachment under load better (Brenner et al., 2020; Carter and Cross, 2005; Ramaiya et al., 2017; Svoboda and Block, 1994). A high load-dependent detachment rate may be due to the fact that KIF1A spends most of its mechanochemical cycle in a one-head-bound state, at least under unloaded conditions (Zaniewski et al., 2020), and provides a mechanism for why KIF1A gives up easily when forced to compete with kinesin-1 motors in driving cargo transport (Arpag et al., 2019; Arpag et al., 2014; Norris et al., 2014). Interestingly, kinesin-2 (KIF3A/KIF3B) and kinesin-5 (Eg5) motors also have a tendency to detach at moderate forces in optical trap assays (Andreasson et al., 2015; Korneev et al., 2007; Milic et al., 2017; Schroeder et al., 2012; Shimamoto et al., 2015; Valentine and Block, 2009; Valentine et al., 2006) and to give up easily when in competition with kinesin-1 (Arpag et al., 2014).

Second, KIF1A motors can only sustain a force up to 3 pN before detachment from the MT track; this is in stark contrast to the ability of kinesin-1 motors to sustain 5–6 pN of force (Brenner et al., 2020; Budaitis et al., 2019; Khalil et al., 2008; Svoboda and Block, 1994). It seems unlikely that the detachment of KIF1A at low forces is due to the strength of the motor–MT interaction, as KIF1A has a higher MT affinity than kinesin-1 in both the ADP-bound (weak MT affinity) and ATP-bound (strong MT affinity and force bearing) states (Atherton et al., 2014; Soppina and Verhey, 2014). It seems more likely that the detachment of KIF1A at low forces can be attributed to a mechanical/structural feature of this motor. An intriguing possibility is that the length of the N-terminal extension that precedes the CS impacts the strength of the CNB and thus the force output of the motor. This possibility is based on recent structural studies and MD simulations of KIF13B which showed that this kinesin-3 motor forms a short CNB with weaker CS–NL interactions than kinesin-1 (Ren et al., 2018a; Ren et al., 2018b). At first glance, previous work on KIF1A’s *C. elegans* homologue, UNC-104, would appear to contradict this model because UNC-104, which also lacks an N-terminal CS extension, frequently generated forces up to 6 pN (Tomishige et al., 2002). However, we have recently determined that these UNC-104 measurements were likely affected by an unintended electronic low-pass filtering of the trapping data so that the reported maximal force of 6 pN is retrospectively estimated to be closer to 4 pN (Brenner et al., 2020). Indeed, when we performed trapping experiments with UNC-104(1–389) using a modern optical tweezers setup and MTs rather than axonemes, we found that UNC-104 stalls at ~3 pN (Fig. 1, B and E; and Fig. S2, A and C). Thus, like KIF1A, single UNC-104 motors sustain lower forces than kinesin-1 motors.

Third, after detachment, KIF1A motors rapidly reattach to the MT and again move forward against the trap. This behavior is dependent on the kinesin-3–specific K-loop (loop 12) whose positively charged residues are responsible for the high landing rate of KIF1A motors (Lessard et al., 2019; Soppina and Verhey, 2014). Indeed, when we replaced the K-loop of KIF1A with the corresponding loop of kinesin-1 (which has only one lysine instead of six), the average number of force generation events per MT encounter was dramatically reduced. Thus, the class-specific K-loop facilitates KIF1A’s ability to rapidly reattach to the MT following detachment, resulting in a characteristic sawtooth pattern for the force versus time plot that has not been observed for other motors to date.

### KAND mutations provide insight into a conserved mechanism of kinesin force generation

Recent structural and biochemical assays with dimeric kinesin-1 motors have provided strong support for the model in which nucleotide-dependent conformational changes in the NL facilitate force generation. NL docking is initiated by an ATP-dependent conformational change in α6 that drives a two-step NL docking: zipping together of the NL’s β9 with the CS (β0) to form the CNB and then latching of the NL’s β10 along the surface of the core motor domain (Budaitis et al., 2019; Hwang et al., 2008; Khalil et al., 2008). Structural studies have shown that similar ATP-induced changes occur to α6 and the NL in members of the kinesin-3 and kinesin-5 families (Atherton et al., 2014; Atherton et al., 2017; Goulet et al., 2012; Goulet et al., 2014; Hesse et al., 2013; Nitta et al., 2008; von Loeffelholz and Moores, 2019), supporting the hypothesis that NL docking is a force-generating mechanism used by all superfamily members. Our work tests this model for a member of the kinesin-3 family.

We focused on two de novo* KIF1A* disease variants, V8M and Y89D, because these residues are predicted to have roles in CNB formation and NL docking based on their (a) location in structural elements of the motor domain associated with force generation in kinesin-1 motors and (b) occurrence in residues that are highly conserved across the kinesin superfamily (Budaitis et al., 2019; Richard et al., 2016). In MD simulations, the V8M and Y89D mutations were predicted to impair docking of the N-terminal (β9) or C-terminal (β10) portions of the NL, respectively, to the KIF1A motor domain. Indeed, using an optical tweezers assay, we found that in the context of minimal dimeric and FL proteins, the V8M and Y89D mutations resulted in a significantly reduced force generation (Fig. 7). The mutations did not, however, affect the ability of the motor to undergo rapid reattachments to the MT (Fig. 3 B). These results extend previous work demonstrating that mutation of β9 results in a decreased velocity in MT-gliding assays (Nitta et al., 2008) to provide evidence that NL docking is critical for force generation by KIF1A motors. More generally, our results extend the model that nucleotide-dependent conformational changes in the NL are an important mechanical element for force generation by kinesin motors.

### KAND mutations relieve autoinhibition, but the active motors display reduced force output, velocity, and processivity

In the context of the FL motor, the V8M and Y89D mutations resulted in a dramatic increase in the number of motility events, indicating that both mutations relieve autoinhibition. Notably, both mutations resulted in an even greater number of motility events than the V483N mutation (Huo et al., 2012), indicating that the V483N mutant is only partially activated. That mutations in the motor domain can relieve autoinhibition has been previously demonstrated for members of the kinesin-1 and kinesin-4 families (Cheng et al., 2014; Kelliher et al., 2018). Our finding that the V8M mutation relieves autoinhibition is consistent with the recent work of Chiba et al. (2019). However, Chiba et al. proposed that the V8M mutation results in hyperactivation of KIF1A based on comparisons with the autoinhibited WT motor. In contrast, we show that the V8M mutant motor is impaired in multiple motility properties when compared with an uninhibited WT motor (Table 3). Therefore, whether the neurodevelopmental disorders in human patients are due to a (toxic) gain of function caused by the relief of autoinhibition, as suggested by Chiba et al. (2019), and/or are the result of reduced force output, velocity, and processivity that result in impaired cargo transport, as suggested in this study, requires further investigation.

Our study shows that the mutation-induced impairments of motor function are multifold. First, KIF1A motors containing V8M or Y89D mutations were significantly impaired in their ability to withstand force in the optical trapping assay. Second, both mutants exhibited significantly reduced velocities and processivities in single-molecule assays, likely due to allosteric effects of NL docking on ATPase activity and MT binding (Figs. 5 and 6; Atherton et al., 2014; Cao et al., 2014; Hahlen et al., 2006; Muretta et al., 2015; Nitta et al., 2008; Shang et al., 2014). Third, both mutants displayed reduced landing rates in the context of the minimal dimeric motor under unloaded conditions. However, the mutations did not affect reattachment to the MT in the optical trapping assay. These results can be explained by an increased ability of the motor to diffuse away upon detaching from the MT in the single-molecule motility assay but to rebind when held close the MT surface in the optical trap. Indeed, a recent paper (Sudhakar et al., 2020) shows that kinesin-1 stays in contact with the MT even when the bead is pulled back to the trap center.

### Effects on cargo transport and implications for disease

The mechanical and motility properties of KIF1A are likely matched to the cellular functions of this motor and are optimized for transport under physiological conditions. KIF1A motors drive long-range transport of synaptic vesicle precursors and dense core vesicles in neurons (Barkus et al., 2008; Hall and Hedgecock, 1991; Lo et al., 2011; Okada et al., 1995; Yonekawa et al., 1998; Zahn et al., 2004) under conditions where teams of two to four motors engage with the MT (Hayashi et al., 2018a; Hayashi et al., 2018b). The fast and superprocessive motility of KIF1A motors would be advantageous for long-distance transport, and a high-force output may not be required for teams of motors to transport small membrane-bound organelles. The rapid detachment and reattachment of individual motors in response to a hindering load would prevent motors from slowing or stalling and thereby help teams of motors navigate obstacles and ensure fast, continuous transport.

How mutations in KIF1A protein cause disease is still unclear, and both loss-of-function and gain-of-function mutations have been linked to human neurodevelopmental and neurodegenerative diseases. V8M and Y89D are de novo mutations that manifest in an autosomal dominant manner. Our results indicate that these mutations result in reduced speed, processivity, landing rate, and force output of single KIF1A motors and delayed transport driven by teams of mutant motors in an unpolarized cell (Fig. 10). The V8M mutation also results in defects in synaptic transmission that manifest in an age-dependent manner (Chiba et al., 2019). Furthermore, our single-molecule motility results suggest that the presence of a mutant motor domain is sufficient to impair the motility properties of heterodimeric WT/mutant motors (Fig. 9). It seems likely that, in patients, transport driven by these mutant motors is compromised, given the long distances and spatial constraints that characterize transport in neuronal cells.

## Materials and methods

### Structural model and MD simulations of KIF1A–motor complex

Initial coordinates of the KIF1A kinesin motor domain in the ATP-bound state (with ATP analogue adenylyl-imidodiphosphate [AMP-PNP]) and in complex with the tubulin heterodimer were taken from PDB accession no. 4UXP (Atherton et al., 2014). The kinesin motor domain sequence was that of *Hs*KIF1A (UniProt identifier Q12756). Missing coordinates, where applicable, were modeled using MODELLER version 9.18 (Šali and Blundell, 1993). A total of 100 models were generated with the following options in MODELLER: variable target function method was set to slow with associated conjugate gradient set to 150 iterations; MD with simulated annealing option was set to slow; and the entire optimization process was repeated twice. The top-scoring model was selected for MD simulations with discrete optimized protein energy score (Shen and Sali, 2006) for loop refinement.

Energy minimization and MD simulations were performed with AMBER 18 (University of California, San Francisco) and the ff99SB AMBER force ﬁeld (Hornak et al., 2006). Nucleotide parameters were obtained from Meagher et al. (2003). Histidine protonation states were assigned based on the their acid dissociation constant values calculated by Propka (Li et al., 2005). MD simulations were started from equilibrated structures with at least four independent runs of at least 200 ns each. All simulations were performed in-house on Nvidia graphics processing unit (GPU) cards with the GPU version of PMEMD (Particle Mesh Ewald Molecular Dynamics; pmemd.cuda). We thank Nvidia for their gift of the GPU card through their academic GPU seed grant. Trajectory analyses were performed in R using the Bio3D v2.3-3 package (Skjærven et al., 2014).

Residue–residue distance differences between WT and the mutant ATP-bound kinesin motor domain in complex with tubulin heterodimer were identified with an ensemble difference distance matrix analysis routine (Budaitis et al., 2019; Muretta et al., 2018). For this analysis, a total of 400 conformations were obtained for each state under comparison by extracting 100 equally time-spaced conformations from the last 20 ns of each simulation replicate. Briefly, the ensemble difference distance matrix routine reduces the difference between long distances, while differences between short distances are kept intact. The significance of residue distance variation between apo and ATP-bound states, and between ATP-bound and mutant states was evaluated with the Wilcoxon test. Residue pairs showing a P value <10^−5^ and an average masked distance difference >1 Å were considered statistically significant residue–residue distance differences for further analysis.

### Plasmids

The FL rat KIF1A corresponds to GenBank accession no. XP_017452403 and was tagged at its C-terminus with mNG for fluorescence microscopy and with an AviTag for biotinylation by coexpressed BirA protein. A constitutively active version (KIF1A(FL^Act^)) was generated by QuickChange introduction of the point mutation V483N (Huo et al., 2012; Soppina et al., 2014). The disease-associated mutations were introduced by Gibson cloning.

The truncated, constitutively active version contains the first 393 amino acids and thus uses its own neck coil sequence for dimerization (Hammond et al., 2009). Because the KIF1A neck coil has a weak propensity to form a stable dimer (Soppina et al., 2014), an LZ sequence was appended after the neck coil to maintain the dimer state. The resulting construct, KIF1A(1–393)-LZ, is referred to simply as KIF1A(1–393) and has been widely used to investigate the motility properties of dimeric KIF1A motors (Guedes-Dias et al., 2019; Karasmanis et al., 2018; Lessard et al., 2019; Monroy et al., 2018; Monroy et al., 2020; Soppina et al., 2014; Soppina and Verhey, 2014). The use of an LZ sequence to maintain the dimer state of truncated kinesin-3 motors has also been documented for the *C. elegans* UNC-104(1–389) and *Drosophila melanogaster* Khc-73 constructs (Huckaba et al., 2011; Tomishige et al., 2002).

For expression in COS-7 cells, KIF1A(1–393) motors were tagged with an AviTag for biotinylation and attachment to beads in optical tweezers assays, with an mNG or Halo-FLAG tag for single-molecule imaging assays, or with mNG-FRB for inducible cargo dispersion assays in cells. For *E. coli* expression, the rat KIF1A(393)-LZ coding sequence was amplified by PCR from KIF1A(393)-LZ-mScarlet-strepII plasmid (gift from Kassandra Ori-McKenney, University of California, Davis, Davis, CA; Monroy et al., 2018). The coding sequence was inserted into pSNAP-tag(T7)-2 vector (New England Biolabs Inc.; N9181S) containing a SNAP_f_-EGFP-6His cassette. Point mutations were generated with the NEB Q5 site-directed mutagenesis kit (New England Biolabs Inc.; E0554S). The KIF1A(393)-LZ-SNAP_f_-EGFP-6His construct was used for the optical tweezers–based force measurements (Fig. 1) and the single-molecule total internal reflection fluorescence (TIRF) motility (Fig. S1) and photobleaching experiments (Fig. S4).

A *Ce*UNC-104(1–389)-LZ-EGFP-6His plasmid (gift from Ron Vale, University of California, San Francisco, San Francisco, CA) was used to generate the UNC-104(1–389)-LZ-HaloTag-6His plasmid by replacing the EGFP coding sequence with that of the HaloTag sequence amplified from pHTC HaloTag CMV-neo vector (Promega; G7711). The original UNC-104(1–389)-LZ-EGFP-6His construct was used for the optical tweezers–based force measurements (Fig. 1 and Fig. S2), while the UNC-104(1–389)-LZ-HaloTag-6His construct was used for the single-molecule TIRF studies (Fig. S1).

The peroxisome-targeting PEX3-mRFP-FKBP construct was a gift from Casper Hoogenraad (Utrecht University, Utrecht, Netherlands; Kapitein et al., 2010). Constructs coding for FRB (DmrA) and FKBP (DmrC) sequences were obtained from ARIAD Pharmaceuticals and are now available from Takara Bio Inc. Plasmids encoding mNG were obtained from Allele Biotechnology and Pharmaceuticals, Inc. Point mutations were generated using QuickChange site-directed mutagenesis. All plasmids were verified by DNA sequencing.

### Cell culture, transfection, and lysate preparation

COS-7 (African green monkey kidney fibroblasts, Research Resource Identifier CVCL_0224; American Type Culture Collection) were grown at 37°C with 5% (vol/vol) CO_2_ in DMEM (Gibco) supplemented with 10% (vol/vol) FetalClone III (HyClone) and 2 mM GlutaMAX (L-alanyl-L-glutamine dipeptide in 0.85% NaCl; Gibco). Cells are checked annually for mycoplasma contamination and were authenticated through mass spectrometry (the protein sequences exactly match those in the African green monkey genome). 24 h after seeding, cells were transfected using TransIT-LT1 transfection reagent (Mirus Bio), and the JF552 HaloTag ligand (Tocris Bioscience) was added to cell culture media to a final concentration of 50 nM. Cells were trypsinized and harvested 24 h after transfection by low-speed centrifugation at 3,000 × *g* at 4°C for 3 min. The pellet was resuspended in cold 1× PBS and centrifuged at 3,000 × *g* at 4°C for 3 min, and the pellet was resuspended in 50 µl of cold lysis buffer (25 mM Hepes/KOH, 115 mM potassium acetate, 5 mM sodium acetate, 5 mM MgCl_2_, 0.5 mM EGTA, and 1% [vol/vol] Triton X-100, pH 7.4) with 1 mM ATP, 1 mM PMSF, and 1% (vol/vol) protease inhibitor cocktail (P8340; Sigma-Aldrich). Lysates were clarified by centrifugation at 20,000 × *g* at 4°C for 10 min, and lysates were snap frozen in 5-µl aliquots in liquid nitrogen and stored at −80°C.

### Protein expression and purification from *E. coli*

Plasmids were transformed into BL21-CodonPlus(DE3)-RIPL competent cells (Agilent Technologies; 230280). A single colony was inoculated in 1 ml of terrific broth with 50 µg/ml carbenicillin and 50 µg/ml chloramphenicol. The 1-ml culture was shaken at 37°C overnight and then inoculated into 400 ml of terrific broth with 2 µg/ml carbenicillin and 2 µg/ml chloramphenicol. The 400-ml culture was shaken at 37°C for 4–5 h and then cooled on ice for 1 h. IPTG was then added to the culture to a final 0.1 mM concentration to induce expression. Afterward, the culture was shaken at 18°C overnight. The cells were harvested by centrifugation at 3,000 relative centrifugal force for 10 min at 4°C. The supernatant was discarded, and 5 ml of B-PER complete bacterial protein extraction reagent (Thermo Fisher Scientific; 89821) with 2 mM MgCl_2_, 1 mM EGTA, 1 mM DTT, 0.1 mM ATP, and 2 mM PMSF, and 10% glycerol was added to the cell pellet to fully resuspend the cells. The resuspended cells were flash frozen and stored at −80°C.

To purify protein, the frozen cells were thawed at 37°C. The solution was nutated at room temperature for 20 min and then dounced for 10 strokes on ice to lyse the cells. The cell lysate was cleared by centrifugation at 80,000 rpm for 10 min at 4°C using a Beckman Coulter tabletop centrifuge unit. The lysate was nutated with 200 µl of Ni-nitrilotriacetic acid resin (Roche cOmplete His-Tag purification resin, 5893682001; MilliporeSigma) at 4°C for 1 h. The resin was washed with wash buffer (50 mM Hepes, 300 mM KCl, 2 mM MgCl_2_, 1 mM EGTA, 1 mM DTT, 1 mM PMSF, 0.1 mM ATP, 0.1% Triton X-100, 10% glycerol, pH 7.2) and labeled with 10 µM SNAP-Cell TMR-Star (New England Biolabs Inc.; S9105S) or HaloTagTMR (Promega; G8251) at room temperature for 10 min. The resin was further washed, and the protein was eluted with elution buffer (wash buffer with 250 mM imidazole). The elute was flash frozen and stored −80°C.

To remove inactive motors, an MT binding and release assay was performed (Rao et al., 2019). 50 µl of eluted protein was buffer exchanged into low-salt buffer (30 mM Hepes, 50 mM KCl, 2 mM MgCl_2_, 1 mM EGTA, 1 mM DTT, and 0.1 mM AMP-PNP) using a 0.5-ml Zeba spin desalting column (7-kD molecular weight cutoff; Thermo Fisher Scientific; 89882). AMP-PNP and Taxol were added to the flow-through to final concentrations of 1 mM and 10 µM, respectively. After 5 µl of 5 mg/ml Taxol-stabilized MTs was added to the mixture, the solution was incubated at room temperature for 5 min to allow motors to bind to the MTs. The mixture was then spun through a 100-µl glycerol cushion (80 mM Pipes, 2 mM MgCl_2_, 1 mM EGTA, 1 mM DTT, 10 µM Taxol, and 60% glycerol) by centrifugation at 40,000 rpm for 10 min at room temperature. Next, the supernatant was removed, and the pellet was resuspended in 50 µl of high-salt buffer (30 mM Hepes, 300 mM KCl, 2 mM MgCl_2_, 1 mM EGTA, 1 mM DTT, 10 µM Taxol, 3 mM ATP, and 10% glycerol). The MTs were then removed by centrifugation at 40,000 rpm for 5 min at room temperature. Finally, the supernatant (i.e., the MT release [MT-R] fraction) was aliquoted, flash frozen in liquid nitrogen, and stored at −80°C.

### TIRF single-molecule motility assays

For motors in COS-7 cell lysates, MTs were polymerized from porcine brain tubulin (T240; Cytoskeleton Inc.) in BRB80 buffer (80 mM Pipes/KOH, pH 6.8, 1 mM MgCl_2_, and 1 mM EGTA) supplemented with GTP and MgCl_2_ and incubated for 60 min at 37°C. 2 µM Taxol in prewarmed BRB80 was added and incubated for 60 min to stabilize MTs. MTs were stored in the dark at room temperature for up to 2 wk. Flow cells were prepared by attaching a 1.5-mm coverslip (Thermo Fisher Scientific) to a glass slide (Thermo Fisher Scientific) using double-sided tape. MTs were diluted in fresh BRB80 buffer supplemented with 10 µM Taxol, infused into flow cells, and incubated for 4 min to allow for nonspecific absorption to the glass. Flow cells were then incubated with blocking buffer (0.5 or 1.0 mg/ml casein in imaging buffer with 10 µM Taxol) for 4 min. Flow cells were then infused with motility mixture (0.5–1.0 µl of COS-7 cell lysate, 25 µl of imaging buffer, 15 µl of blocking buffer, 1 mM ATP, 0.5 µl of 100 mM DTT, 0.5 µl of 20 mg/ml glucose oxidase, 0.5 µl of 8 mg/ml catalase, and 0.5 µl of 1 M glucose). To optimize the single-molecule imaging conditions for KIF1A motors in COS-7 cell lysates, the following imaging buffers were tested: P12 (12 mM Pipes/KOH, pH 6.8, 1 mM MgCl_2_, and 1 mM EGTA), BRB40 (40 mM Pipes/KOH, pH 6.8, 1 mM MgCl_2_, and 1 mM EGTA), BRB80 (80 mM Pipes/KOH, pH 6.8, 1 mM MgCl_2_, and 1 mM EGTA), or PERM (25 mM Hepes/KOH, 115 mM potassium acetate, 5 mM sodium acetate, 5 mM MgCl_2_, and 0.5 mM EGTA, pH 7.4). To optimize the amount of KIF1A motor for distinguishing and tracking single motors, the amount of lysate added to the motility mixture was varied and optimized for each motor. For homodimeric truncated KIF1A(1–393) motors, the final concentrations were as follows: WT/WT, 0.65–1.27 nM; V8M/V8M, 3.23–8.73 nM; and Y89D/Y89D, 1.05–4.90 nM. For heterodimeric truncated KIF1A(1–393) motors, the final concentrations were as follows: WT/WT, 1.89–10.54 nM; WT/V8M, 0.31–9.94 nM; and WT/Y89D, 2.32–11.48 nM. For FL KIF1A, the final concentrations of all motors were set at 0.3 nM, and 5 mg/ml casein was included in the motility mixture. The flow cells were sealed with molten paraffin wax and imaged on an inverted Nikon Ti-E/B TIRF microscope with a perfect focus system; a 100×, 1.49 NA oil immersion TIRF objective; three 20-mW diode lasers (488 nm, 561 nm, and 640 nm); and an EM charge-coupled device camera (iXon+ DU879; Andor). Image acquisition was controlled using Nikon Elements software, and all assays were performed at room temperature (25°C). Images were acquired at 100 ms per frame for 200 frames.

Single-molecule TIRF motility studies of *E. coli*–expressed and purified KIF1A(1–393)-LZ-SNAP_f_-EGFP-6His and UNC-104(1–389)-LZ-HaloTag-6His labeled with TMR ligand were performed as previously described (Rao et al., 2019). Motility assays were performed in BRB40 buffer with MTs 10–35 µm in length. For each movie, a total of 600 frames was acquired with an acquisition time of 100 or 200 ms per frame.

Motility data were analyzed by first generating maximum-intensity projections to identify MT tracks and then generating kymographs (segmented line with width = 3 pixels; Fiji/ImageJ). All motility events that lasted more than three frames (>300 ms) were analyzed and identified as diffusive or processive. Diffusive events were defined as those with forward-and-backward motion and net displacement <200 nm. Processive events were defined as those with net displacement ≥200 nm in one direction. Landing rates were calculated as the number of processive events per second per nanomolar motor per micrometer of MT, and an unpaired Student’s *t* test was used to assess whether the distributions were significantly different between motors. The processive events were then further analyzed to calculate velocities and run lengths, and the data were plotted as histograms. At least 150 processive events were quantified for each motor using at least three independent lysate preparations and three or more independent trials. Motor velocities were fitted to a Gaussian cumulative distribution as previously described (Norris et al., 2014), and an unpaired Student’s *t* test was used to assess whether the distributions were significantly different between motors.

Motor run lengths were fit using a Gaussian or exponential distribution based on rate and shape parameters derived from fitting cumulative distributions (Norris et al., 2014). Median values with percentiles (25%, 75%) were calculated, and a Kruskal-Wallis test was used to assess whether the distributions were significantly different between motors. All KIF1A motility events were included, including those that end when the motor reaches the end of an MT; thus, the reported run lengths are an underestimation of the motor’s processivity. Calculating run lengths for superprocessive motors is limited by several factors. First, the characteristic run length measured in an experiment depends on the underlying distribution of MT track lengths in the imaging chamber (Ruhnow et al., 2017; Thompson et al., 2013). For truncated KIF1A(1–393) motors in COS-7 lysates (e.g., KIF1A(1–393)^C^), the motility assays were performed with MTs 35–75 µm in length, whereas for FL KIF1A motors in COS-7 lysates (e.g., KIF1A(FL)^C^), the MTs were 5–55 µm in length. Histograms of MT lengths were generated for each motor to ensure that the different motors were provided with similar MT tracks. The measured run lengths are also affected by the buffer conditions of the motility assay and the fluorescent tag on the motor (Fig. S5; Norris et al., 2015).

### Optical tweezers assay

The polystyrene trapping beads, MTs, and slides were prepared as described previously (Rao et al., 2019). Briefly, Polybead carboxylate microspheres with an average diameter of 520 nm (Polyscience Inc.; 09836-15) were coated with streptavidin and α-casein or with an anti-GFP antibody and α-casein. Coverslips (Carl Zeiss Microscopy; 474030-9000-000) were cleaned with 25% HNO_3_ and 2 M NaOH, washed with double-distilled H_2_O, air dried, and stored at 4°C. The flow chamber was assembled with a glass slide, parafilm stripes, and a cleaned coverslip as described previously (Rao et al., 2019). MTs with incorporated biotinylated tubulin were attached to the coverglass surface via α-casein-biotin and streptavidin. Control cell lysate without KIF1A expression was tested to ensure there were no nonspecific interactions between other endogenous motors in the lysate and the beads. 100 beads were tested, and no force generation was observed for the control cell lysate under the experimental conditions used for cell lysates containing tagged KIF1A constructs. Inactive motors in cell lysate with KIF1A were removed via an MT binding and release step as described for the *E. coli*–expressed motors. The MT-R fraction from COS-7 lysates was prediluted 50–200×, while the MT-R fraction of *E. coli*–expressed KIF1A was prediluted 200–5,000×. 1 µl of the predilution was incubated with 0.4-µl beads on ice for 15 min. For experiments with the cell lysate, the lysate was prediluted so that <10% of the beads showed force generation; for experiments with the *E. coli*–expressed KIF1A, the solution was diluted so that <30% of the beads tested showed force-generation events. Finally, the protein–bead mixture was diluted in 40 µl of assay buffer (60 mM Hepes, 50 mM KAc, 2 mM MgCl_2_, 1 mM EGTA, 1 mM DTT, 10 µM Taxol, 2 mM ATP, 50 mM glucose, gloxy, 0.75 mg/ml α-casein, and 10% glycerol) and flowed into the slide chamber. All optical trapping experiments were performed with a LUMICKS C-Trap. Motor steps (approximate center-of-mass movement of UNC-104^E^ and KIF1A^E^; Fig. 1 H and Fig. S3, E and F) were determined from the bead displacement records using a step-finding algorithm developed by Kerssemakers et al. (2006).

### Inducible peroxisome dispersion assay

The inducible peroxisome dispersion assay is based on previous work (Budaitis et al., 2019; Efremov et al., 2014; Kapitein et al., 2010; Schimert et al., 2019). Plasmids for expression of WT or mutant rat KIF1A(1–393) motors tagged with mNG and an FRB domain were cotransfected into COS-7 cells with a plasmid for expression of PEX3-mRFP-FKBP at a ratio of 6:1 with TransITLT1 transfection reagent (Mirus Bio). 8 h after transfection, rapamycin (Calbiochem/MilliporeSigma) or ethanol vehicle was added to a final concentration of 44 nM to promote FRB and FKBP heterodimerization and recruitment of motors to peroxisomes. 5, 10, or 30 min after addition of rapamycin and recruitment of motors to the surface of peroxisomes, cells were fixed with 3.7% formaldehyde (Thermo Fisher Scientific) in 1× PBS for 10 min, quenched in 50 mM ammonium chloride in PBS for 5 min, and permeabilized in 0.2% Triton X-100 in PBS for 5 min. Coverslips were mounted in ProLong Gold (Invitrogen) and imaged using an inverted epifluorescence microscope (Nikon TE2000E) with a 40×/0.75 NA objective and a CoolSnapHQ camera (Photometrics). Only cells expressing low levels of motor-mNG-FRB were imaged and included in quantification. The phenotype of cargo dispersion was scored as clustered, partial dispersion, diffuse dispersion, or peripheral dispersion based on the signal localization in the PEX3-mRFP-FKBP (peroxisome) channel. The data for each construct across three independent trials is summarized as a stacked bar plot.

### Statistical analysis

The statistical tests applied to each set of data are described in the corresponding text, figure or table legends, and Materials and methods sections. For each dataset, three independent replicates were quantified unless stated otherwise. For velocity and landing rate measurements, the data were assumed to be normally distributed, but this was not formally tested, and statistical differences between mean values were determined using an unpaired Student’s *t* test. For run-length measurements, the datasets include both normally and not-normally distributed data, and statistical significance between median values was determined using a nonparametric Kruskal-Wallis test. For all other datasets, the data were assumed to be normally distributed, but this was not formally tested, and the differences between mean values were analyzed using an unpaired Welch’s *t* test. All statistical analyses were performed using GraphPad Prism software (version 8.0).

### Primers for cloning and mutagenesis

The primers used for cloning and mutagenesis were as follows: NDEI-KIF1A-F, 5′-GAG​ATA​TAC​ATA​TGG​CTG​GGG​CCT​CTG​TGA-3′; ECORI-KIF1A-F, 5′-TCC​ATG​GTG​AAT​TCA​ACT​AAT​TTC​TTT​AAT​CTG​GCA​ACC​TCA​TTT​TCC-3′; V8M-F, 5′-CTC​TGT​GAA​GAT​GGC​GGT​GCG-3′; V8M-R, 5′-GCC​CCA​GCC​ATA​TGT​ATA​TCT​CCT​TC-3′; Y89D-F, 5′-CTT​TGA​GGG​CGA​CAA​CGT​GTG​CAT​C-3′; Y89D-R, 5′-GCA​TGC​TGT​AGC​ATC​TCC​TCC​CC-3′; SW-F, 5′-AAA​ACC​TTC​ATC​CCT​TAC​CGA​GAC​TCG​GTA​TTG-3′; SW-R, 5′-AGT​ACC​TTC​AGC​GAG​AGC​AGA​GAT​GAC​CTT​TC-3′; SWA-F, 5′-GCT​ACC​TTC​ATC​CCT​TAC​CGA​GAC​TCG​GTA​TTG-3′; SWA-R, 5′-AGT​ACC​TTC​AGC​GAG​AGC​AGA​GAT​GAC​CTT​TC-3′; V483N-F, 5′-GCC​CTG​CTG​GCC​GAG​ATG​GGT​AAC​GCC​ATG​AGG​GAA​GAT​GGT​GGC-3′; and V483N-R, 5′-GCC​ACC​ATC​TTC​CCT​CAT​GGC​GTT​ACC​CAT​CTC​GGC​CAG​CAG​GGC-3′.

### Online supplemental material

Fig. S1 shows single-molecule motility data for the *E. coli*–expressed and TMR-labeled motors UNC-104(1–389), KIF1A(1–393), KIF1A(1–393)-V8M, and KIF1A(1–393)-Y89D. Fig. S2 depicts the analysis of UNC-104 generation in BRB12 buffer. Fig. S3 shows the results of additional stall force, step size, and cluster length analyses for KIF1A(1–393) and KIF1A(FL^Act^) motors. Fig. S4 shows the photobleaching analysis of TMR-labeled KIF1A(1–393)^E^ WT and mutants. Fig. S5 shows the influence of fluorescence tag and buffer conditions on KIF1A(1–393) motility and strategies for designing heterodimeric motors.
